# Neutrophil‐Targeting Semiconducting Polymer Nanotheranostics for NIR‐II Fluorescence Imaging‐Guided Photothermal‐NO‐Immunotherapy of Orthotopic Glioblastoma

**DOI:** 10.1002/advs.202406750

**Published:** 2024-08-19

**Authors:** Jiansheng Liu, Danling Cheng, Anni Zhu, Mengbin Ding, Ningyue Yu, Jingchao Li

**Affiliations:** ^1^ State Key Laboratory for Modification of Chemical Fibers and Polymer Materials College of Biological Science and Medical Engineering Donghua University Shanghai 201620 China

**Keywords:** cancer theranostics, fluorescence imaging, glioblastoma, immunotherapy, semiconducting polymer

## Abstract

Glioblastoma (GBM) is one of the deadliest primary brain tumors, but its diagnosis and curative therapy still remain a big challenge. Herein, neutrophil‐targeting semiconducting polymer nanotheranostics (SSPN_iNO_) is reported for second near‐infrared (NIR‐II) fluorescence imaging‐guided trimodal therapy of orthotopic glioblastoma in mouse models. The SSPN_iNO_ are formed based on two semiconducting polymers acting as NIR‐II fluorescence probe as well as photothermal conversion agent, respectively. A thermal‐responsive nitric oxide (NO) donor and an adenosine 2A receptor (A2AR) inhibitor are co‐integrated into SSPN_iNO_ to enable trimodal therapeutic actions. SSPN_iNO_ are surface attached with a neutrophil‐targeting ligand to mediate their effective delivery into orthotopic GBM sites via a “Trojan Horse” manner, enabling high‐sensitive NIR‐II fluorescence imaging. Upon NIR‐II light illumination, SSPN_iNO_ effectively generates heat via NIR‐II photothermal effect, which not only kills tumor cells and induces immunogenic cell death (ICD), but also triggers controlled NO release to strengthen tumor ICD. Additionally, the encapsulated A2AR inhibitor can modulate immunosuppressive tumor microenvironment by blocking adenosine‐A2AR pathway, which further boosts the antitumor immunological effect to observably suppress the orthotopic GBM progression. This study can provide a multifunctional theranostic nanoplatform with cumulative therapeutic actions for NIR‐II fluorescence imaging‐guided effective GBM treatment.

## Graphical abstract

Neutrophil‐targeting semiconducting polymer nanotheranostics are designed for second near‐infrared fluorescence imaging‐guided combination therapy of orthotopic glioblastoma. The nanotheranostics can cross blood‐brain barrier and accumulate in tumor sites via hijacking neutrophils, which enables heat generation via photothermal effect to release nitric oxide and adenosine 2A receptor inhibitor to modulate tumor microenvironment. The nanotheranostics thus enable imaging‐guided photothermal‐NO‐immunotherapy to treat orthotopic glioblastoma.

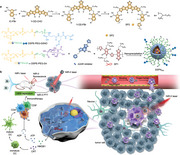

## 1 Introduction

Glioblastoma (GBM) is a common malignant brain tumors originating from glia cells, with an annual incidence of 3–4/100 000, accounting for ≈49% of all gliomas.^[^ ^1^ ^]^ The standard treatment algorithm for GBM typically involves maximum surgical resection followed by a combination of radiotherapy and chemotherapy.^[^ ^2^ ^]^ Due to the infiltrative growth pattern and heterogeneous nature of GBM, this therapeutic strategy fails to achieve significant improvements in the prognosis of patients with GBM, with a devastatingly low median survival of <21 months.^[^ ^3^ ^]^ Therefore, it is urgent to develop novel treatment strategies for GBM. Immunotherapy has attracted considerable attention in the realm of GBM treatment via effectively eliminating cancer cells and inducing immune memory to prevent recurrence.^[^ ^3^ ^]^ However, the efficacy of immunotherapy against GBM is significantly compromised by the immunosuppressive tumor microenvironment and the formidable barrier presented by the blood‐brain barrier (BBB).^[^ ^4^ ^]^ As an immunologically cold tumor, GBM exhibits a tumor microenvironment marked by low numbers of tumor‐infiltrating lymphocytes and high levels of immunosuppressive cells.^[^ ^5^ ^]^ Furthermore, the activities of effector immune cells including dendritic cells (DCs), cytotoxic T lymphocytes (CTLs) and anti‐tumor macrophages were inhibited by immunosuppressive tumor microenvironment.^[^ ^6^ ^]^ This “cold” phenotype of GBM could be partially reversed by treament‐induced immune regulatory effects.^[^ ^3^, ^7^ ^]^ For instance, certain chemotherapeutic agents, such as mitoxantrone, have been shown to trigger immunogenic cell death (ICD) in tumor cells, which leads to DCs maturation and subsequent activation of CTLs.^[^ ^8^ ^]^ However, potential side effects of chemotherapy still remain, and some more safe ICD‐inducing strategies should be explored to enhance the immunotherapy of GBM.

Photothermal therapy (PTT) has substantial promise in the therapeutics of various cancers including GBM.^[^ ^9^ ^]^ Particularly, PTT in the second near‐infrared (NIR‐II) biowindow (1000–1700 nm) is uniquely advantageous for treatments of deep‐seated brain tumors owing to the enhanced penetration ability of NIR‐II light (3–5 cm of depth).^[^ ^10^ ^]^ In addition to killing tumor cells directly via photothermal effect, PTT can promote the anti‐tumor immune response against GBM via triggering ICD effect and activating immune cells.^[^ ^11^ ^]^ However, the PTT‐induced ICD is often weak because of the thermoresistance of tumor cells and potential thermal damages to nearby normal cells.^[^ ^12^ ^]^ Thus, the integration of PTT with other modalities such as gas therapy has been proposed to strengthen immunotherapy.^[^ ^13^ ^]^ Nitric oxide (NO) has emerged as a promising modulator for the immunosuppressive tumor microenvironment as a gaseous signaling molecule.^[^ ^14^ ^]^ Previous studies have demonstrated that NO can prompt a transition in macrophage polarization, shifting them from an immunosuppressive M2 phenotype to antitumorigenic M1 phenotype with enhanced infiltration of T cell and decreased expression of programmed death‐ligand 1,^[^ ^14^, ^15^ ^]^ and potently trigger ICD effect.^[^ ^16^ ^]^ Therefore, NO therapy has been widely used to potentiate cancer immunotherapy.^[^ ^17^ ^]^ Although a synergistic NO and PTT therapy strategy has demonstrated high anti‐tumor efficacy,^[^ ^18^ ^]^ their combination to sensitize immunotherapy for GBM treatment has been rarely explored.

The integrations of imaging capability into therapeutic platforms have garnered increasing interests in oncotherapy as the treatment precision can be significantly enhanced.^[^ ^19^ ^]^ Although clinical imaging approaches such as magnetic resonance have improved the detection rates of GBM, these imaging modalities still suffer from insufficient temporal and spatial resolution for tumor delineation, as well as potentially harmful ionizing radiation.^[^ ^20^ ^]^ These drawbacks greatly affect their capacities to delineate the infiltrative glioma tissues from surrounding healthy brain tissues.^[^ ^21^ ^]^ Alternatively, fluorescence imaging is notable due to its high sensitivity and selectivity, making it advantageous for intraoperatively visualizing boundary between glioma and healthy tissues.^[^ ^22^ ^]^ Specifically, NIR‐II fluorescence imaging has the merits of deep tissue penetration, robust spatiotemporal resolution, and enhanced imaging contrast, thus holding great promise for GBM detection.^[^ ^23^ ^]^ Considering the obvious advantages, it is necessary to explore the possibilities of NIR‐II fluorescence imaging‐guided combinational therapy of GBM.

**Figure 1:**
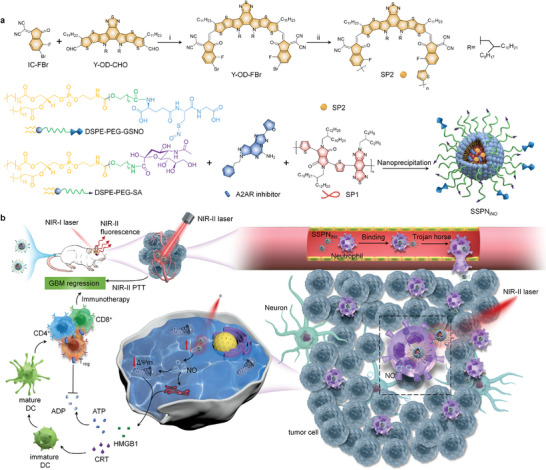
Scheme of nanotheranostics for imaging‐guided photothermal‐NO‐immunotherapy of orthotopic GBM. a) Synthesis of SP2 and nanotheranostics (SSPN_iNO_). i) Pyridine, CHCl_3_, 65 °C, 30 min; ii) thiophene‐tin, Pd_2_(dba)_3_, P(o‐tol)_3_, toluene, 110 °C, 72 h. b) Mechanism scheme of SSPN_iNO_‐based theranostics of GBM.

In this study, semiconducting polymer (SP)‐based nanotheranostics were designed for NIR‐II fluorescence imaging‐guided photothermal‐NO‐immunotherapy of orthotopic GBM. To increase GBM targeting capability, a neutrophil‐targeting ligand sialic acid (SA) was covalently attached to the nanotheranostics (**Figure** **1**a). Neutrophils can traverse BBB and penetrate into glioma tumors and thus the SA conjugation could mediate BBB crossing and improved delivery efficacy of nanotheranostics into GBM sites via binding onto the surface of neutrophils.^[^ ^24^ ^]^ These nanotheranostics contained two SPs to enable NIR‐II fluorescence imaging and NIR‐II PTT of GBM under NIR‐II laser irradiation, respectively. The generated heat killed tumors cells and triggered the release of NO from thermal‐responsive NO donor to achieve PTT‐gas therapy, sensitizing GBM tumors via inducing ICD effect (Figure 1b). Such nanotheranostics delivered an adenosine 2A receptor (A2AR) inhibitor to modulate immunosuppressive microenvironment of GBM, further boosting antitumor immune responses. As such, NIR‐II fluorescence imaging‐guided photothermal‐NO‐immunotherapy showed a high efficacy for GBM treatment.

## 2 Results and Discussion

### 2.1 Nanotheranostic Characterization

A thermal‐responsive NO donor S‐nitrosoglutathione (GNSO) was conjugated to DSPE‐PEG‐NHS via amidation reaction to obtain DSPE‐PEG‐GNSO (Figure S1, Supporting information). The UV–vis spectrum of DSPE‐PEG‐GNSO exhibited a distinct 330 nm absorption peak (Figure S2, Supporting information), indicative of the π → π^*^ electronic transitions associated with SNO group.^[^ ^16^, ^25^ ^] 1^H NMR was also employed to verify the formation of DSPE‐PEG‐GNSO, showing that the majority of resonance peaks of NHS group diminished, while the resonance peak associated with GSNO at ≈4.5 ppm could be detected (Figure S3, Supporting information). A neutrophil‐targeting ligand (SA) was conjugated to amphiphilic polymer to synthesize DSPE‐PEG‐SA (Figure S4, Supporting information) and the successful conjugation was confirmed via ^1^H NMR (Figure S5, Supporting information). To enable NIR‐II fluorescence imaging, a novel fluorescent SP2 was synthesized (Figure S6, Supporting information). The intermediate products and final SP2 were validated using ^1^H and ^13^C NMR (Figures S7–S11, Supporting information). The gel permeation chromatography analysis determined the molecular weight of SP2 to be 29 300 Da. SP2 exhibited robust absorption within the NIR‐I range and emitted fluorescence within the NIR‐II spectrum (Figure S12, Supporting information). The objective nanotheranostics (SSPN_iNO_) were prepared via nanoprecipitation of SP1, SP2, A2AR inhibitor, DSPE‐PEG‐GSNO and DSPE‐PEG‐SA at an optimal feeding mass ratio of 0.5:0.2:1.0:5:20. The encapsulation efficiency and loading capacity for A2AR inhibitor was 62.9% and 2.5%, respectively. Non‐targeted nanoparticles (SPN_iNO_) were constructed using SP1, SP2, A2AR inhibitor, DSPE‐PEG‐GSNO and DSPE‐PEG in a consistent feeding mass ratio. In addition, counterpart nanotheranostics without A2AR inhibitor loading (SSPN_NO_) were synthesized via the similar route.

**Figure 2:**
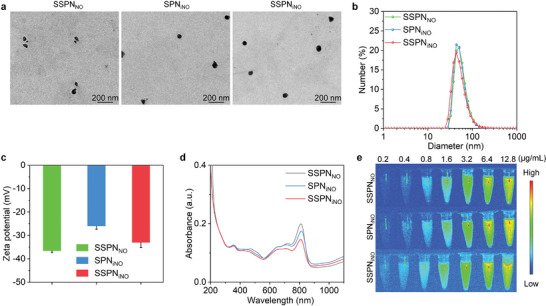
Characterization of nanotheranostics. a) Images of SSPN_NO_, SPN_iNO_, and SSPN_iNO_ using transmission electron microscope. b) Size distribution of SSPN_NO_, SPN_iNO_, and SSPN_iNO_ via DLS analysis. c) Zeta potentials of SSPN_NO_, SPN_iNO_, and SSPN_iNO_ in solution (*n* = 5). Data are expressed as mean ± SD. d) UV‐vis‐NIR spectra of SSPN_NO_, SPN_iNO_ and SSPN_iNO_. e) NIR‐II fluorescence images of SSPN_NO_, SPN_iNO_, and SSPN_iNO_ at different concentrations.

All these nanoparticle formations (SSPN_iNO_, SPN_iNO_, and SSPN_NO_) displayed a spherical morphology (**Figure** **2**a). Dynamic light scattering (DLS) measurements of SSPN_iNO_, SPN_iNO_, and SSPN_NO_ revealed their comparable particle size of ≈55.0 nm (Figure 2b). The particle sizes remained consistent after different incubation periods, suggesting excellent stability of the as‐prepared nanoparticles (Figure S13, Supporting information). Zeta potentials were −37.1, −26.2, and −33.5 mV for SSPN_NO_, SPN_iNO_, and SSPN_iNO_, respectively (Figure 2c). Due to the loadings of SP1 and SP2, these nanoparticles showed similar and strong absroption in the NIR‐II region (Figure 2d). Additionally, SSPN_NO_, SPN_iNO_, and SSPN_iNO_ showed good NIR‐II fluorescence imaging performance in a concentration‐dependent fashion (Figure 2e; Figure S14, Supporting information). Since the fluorescence emission of SP1 was not detected (Figure S15, Supporting information), the robust fluorescence imaging performance of nanotheranostic should be ascribed to the loaded SP2. The findings revealed that the formed three nanoparticles had similar size, surface potential, and optical property.

**Figure 3:**
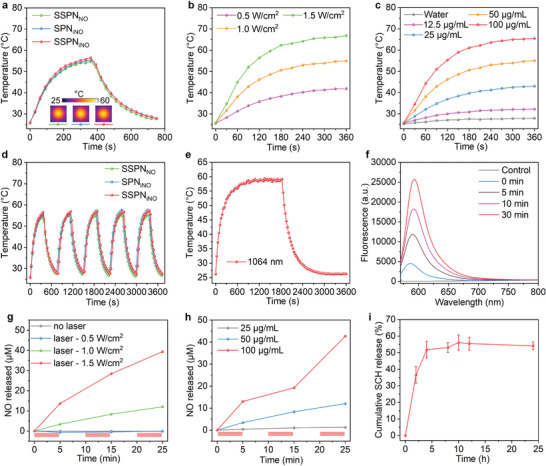
NO and A2AR inhibitor releasing property evaluation. a) Temperature curves of SSPN_NO_, SPN_iNO_, and SSPN_iNO_ irradiated with 1064 nm laser. Inset: infrared thermal images after laser irradiation for 360 s. b) Temperature curves of SSPN_iNO_ solution exposed to NIR‐II laser irradiation at different power intensities. c) Temperature curves of SSPN_iNO_ solutions at different concentrations upon laser irradiation. d) Heating and cooling temperature curves of SSPN_NO_, SPN_iNO_, and SSPN_iNO_ after five on/off cycles of laser irradiation. e) Temperatures of SSPN_iNO_ solution irradiated with NIR‐II laser for 30 min and cooled naturally. f) The fluorescence spectra of NO fluorescence probe (λ_ex_ = 560 nm) and SSPN_iNO_ after irradiation for different time periods. g) NO release behaviors of SSPN_iNO_ under NIR laser irradiation (red rectangles) at different laser power intensities. h) NO release behaviors of SSPN_iNO_ at different concentrations under NIR laser irradiation (red rectangles). i) Cumulative release of A2AR inhibitor from SSPN_iNO_ in aqueous solution (*n* = 5). Data are expressed as mean ± SD.

To investigate the photothermal performance of as‐prepared nanotheranostics, temperature fluctuations in nanoparticle solutions exposed to 1064 nm laser illumination were monitored using infrared thermal camara. The temperatures of SSPN_NO_, SPN_iNO_, and SSPN_iNO_ solutions increased rapidly under laser irradiation and reached peak (55 °C) after 6 min of illumination (**Figure** **3**a). The heating and cooling curves of these nanoparticles were similar, suggesting their consistent photothermal performances. The photothermal effects of nanotheranostics exhibited a dependence on both the nanoparticle concentration and laser power intensity. Upon 6‐min laser irradiation, the temperature of SSPN_iNO_ solution increased to 41.9, 55.0, and 66.9 °C for the laser power intensity of 0.5, 1.0, and 1.5 W cm^−2^, respectively (Figure 3b). When the power intensity was set at 1.0 W cm^−2^, the temperature of SSPN_iNO_ solution increased to 32.2, 43.0, 55.0, and 65.5 °C at concentration of 12.5, 25, 50, and 100 µg mL^−1^, respectively (Figure 3c). During 5 cycles of NIR‐II laser on/off, the heating and natural cooling curves almost unchanged for SSPN_NO_, SPN_iNO_, and SSPN_iNO_ (Figure 3d), suggesting their good photothermal stability. The photothermal conversion efficiency of these nanoparticles was ≈57.4% (Figure 3e; Figure S16, Supporting information).

NO could be released from thermal‐responsive NO donor after photothermal‐triggered S─NO bond cleavage.^[^ ^16^, ^26^ ^]^ A NO fluorescent probe was utilized to confirm the production of NO following laser irradiation.^[^ ^16^ ^]^ NIR‐II laser irradiation led to a significant increase in fluorescence intensities of NO fluorescent probe, with the magnitude of this enhancement positively correlating with the irradiation duration (Figure 3f). The photothermal‐triggered NO release was quantitatively investigated by Griess assay. The release of NO was negligible without laser irradiation, which however exhibited dependence on both the intensity of laser power and the concentration of nanoparticles under laser irradiation. Laser at 0.5 W cm^−2^ was insufficient to elicit NO release, while laser at 1.5 W cm^−2^ could trigger significantly more NO release as compared with that of 1.0 W cm^−2^ (Figure 3g). The concentration of SSPN_iNO_ had a direct proportional effect on the amount of NO release under identical laser irradiation conditions (Figure 3h). The release of A2AR inhibitor was also evaluated and the results showed that ≈60% of drugs could be released in 24 h (Figure 3i).

### 2.2 Evaluation of Nanotheranostic Effects in Cells

**Figure 4:**
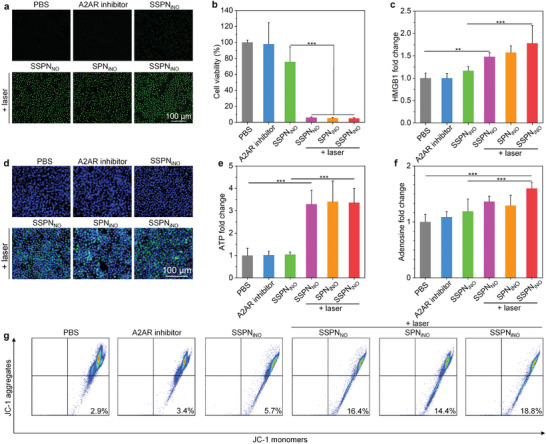
NO generation, therapeutic effect, and DAMP release evaluation. a) NO staining of the treated C6 cells. b) Cell viability of the treated C6 cells (*n* = 6). c) Extracellular HMGB1 release from C6 cells following various treatments (*n* = 5, *p* = 0.0073 for PBS vs SSPN_NO_ + laser; *p* = 0.0004 for SSPN_iNO_ vs SSPN_iNO_ + laser). d) CRT expression of C6 cells after different treatments. e) Extracellular ATP levels from different treated C6 cells (*n* = 5). f) Extracellular adenosine levels from different treated C6 cells (*n* = 5, *p* = 0.0009 for SSPN_iNO_ vs SSPN_iNO_ + laser). g) Analysis of JC‐1 monomers and aggregates in C6 cells exposed to different treatments. Data are presented with mean ± SD (^*^ *p* < 0.05, ^**^ *p* < 0.01, ^***^ *p* < 0.001, ANOVA with Turkey's post‐hoc tests, for *p* without exact value, *p* < 0.0001).

C6 glioma cells were incubated with SSPN_NO_, SPN_iNO_ and SSPN_iNO_ and irradiated by the NIR‐II laser to evaluate the photothermal‐triggered NO release using a fluorescence NO probe. Significant amounts of intracellular NO were generated in both nanoparticles and laser‐treated cells (**Figure** **4**a). In contrast, incubation with SSPN_iNO_ without laser irradiation only elicited a slight increase in intracellular NO level (Figure S17, Supporting information), which was potentially attributed to the minimal spontaneous hydrolysis of GSNO.^[^ ^27^ ^]^ These data indicated that NO was released from nanoparticles in a light‐controlled manner at cellular level.

The in vitro therapeutic efficacy of SSPN_NO_, SPN_iNO_, and SSPN_iNO_ was investigated using C6 tumor cells. The cell viability of C6 cells was 86.8%, 85.6%, and 78.8% for 50 µg mL^−1^ of SSPN_NO_, SPN_iNO_, and SSPN_iNO_, respectively (Figure S18, Supporting information), indicating negligible cytotoxicity of these nanoparticles without laser irradiation. When subjected to laser, the viability of C6 cells was markedly reduced to ≈6.0% for all the nanoparticle‐treated cells, while free A2AR inhibitor (10 µg mL^−1^) did not exhibit significant cytotoxicity toward C6 cells (Figure 4b). Similar therapeutic effects of these nanoparticles with laser irradiation were found via live/dead staining analysis (Figures S19 and S20, Supporting information). These results verified the good therapeutic efficacy of nanotheranostic‐mediated PTT and NO gas therapy upon NIR‐II irradiation.

Apart from their therapeutic efficacies, PTT and NO gas therapy also have the potential to elicit ICD, marked by the release of danger‐associated molecular patterns (DAMPs), such as high mobility group box 1 (HMGB1), calreticulin (CRT) and adenosine triphosphate (ATP).^[^ ^28^ ^]^ The expression of these ICD markers were evaluated using C6 cells. The extracellular levels of HMGB1 exhibited no significant differences for C6 cells treated with PBS, free A2AR inhibitor and SSPN_iNO_ (Figure 4c). When laser was applied in the presence of nanotheranostics, however, the HMGB1 release was significantly increased. Under NIR‐II laser irradiation, C6 cells after SSPN_iNO_ treatment exhibited an ≈1.5‐fold increase in the HMGB1 release compared to control cells, which were simialr to the cells in SSPN_NO_ + laser and SPN_iNO_ + laser groups. CRT staining also showed significantly increased expression after treatments with these nanotheranostics and NIR‐II laser irradiation (Figure 4d). The fluorescence intensity of CRT in C6 cells after treatments with SSPN_NO_, SPN_iNO_, and SSPN_iNO_ and NIR‐II laser exhibited a substantial increase, rising by 20.0‐fold compared to that of the control cells (Figure S21, Supporting information). Consistently, laser irradiation of SSPN_NO_‐, SPN_iNO_‐ and SSPN_iNO_‐treated cells resulted in significantly higher ATP levels (≈3.5‐fold) as compared to that in the control cells (Figure 4e). The released ATP after treatments could be converted to adenosine by ectonucleotidases in tumor microenvironment.^[^ ^29^ ^]^ Thus, the extracellular adenosine level for C6 cells after SSPN_iNO_ treatment and NIR‐II laser irradiation exhibited a 1.6‐fold increase compared to the control group (Figure 4f). To explore the mechanism underlying DAMPs release, different forms of 5,5′,6,6′‐tetrachloro‐1,1′,3,3′‐tetraethylbenzimidazolcarbocyanine iodide (JC‐1) in C6 cells after treatments were analyzed. JC‐1 will aggregate under physiological conditions and exist as monomer if there is a collapse in the mitochondrial membrane potential (ΔΨ_m_).^[^ ^16^ ^]^ As expected, laser irradiation of SSPN_NO_‐, SPN_iNO_‐ and SSPN_iNO_‐treated C6 cells significantly increased the ratios of JC‐1 monomers by ≈5.0‐fold (Figure 4g). These results indicated that cancer cells after nanotheranostics and NIR‐II laser treatments could robustly induce ICD by upregulating HMGB1, CRT, and ATP. A loss of ΔΨ_m_ as demonstrated by increased ratio of JC‐1 monomers suggested that mitochondrial outer membrane permeabilization played a vital role in triggering DAMP releases.^[^ ^16^, ^30^ ^]^

### 2.3 Brain Targeting and Fluorescence‐Imaging Capability of Nanotheranostics

The BBB in GBM greatly limites the accumulation of nanoparticles into tumor sites, thus compromising the therapeutic efficacies.^[^ ^31^ ^]^ To investigate BBB penetration capability of these nanotheranostics, fluorescence dye chlorin e6 (Ce6) was used to label SSPN_NO_, SPN_iNO_, and SSPN_iNO_. UV–vis and fluorescence spectra validated the successful loading of Ce6 into these nanotheranostics (Figure S22, Supporting information). Cellular uptake of Ce6‐labeled nanotheranostics by C6 cells, RAW 264.7 macrophages, and neutrophils were investigated using flow cytometry (Figure S23, Supporting information). The fluorescence intensity of C6 cells was increased by 9.0‐fold after incubation with Ce6‐labeled SSPN_NO_ and SSPN_iNO_, while which was increased by ≈11.0‐fold for macrophages. In contrast, SSPN_NO_ and SSPN_iNO_ showed a significantly enhanced cellular uptake by neutrophils (≈24.0‐fold) as compared to SPN_iNO_ (≈15.0‐fold). These verified that SSPN_NO_ and SSPN_iNO_ could be preferentially endocytosed by neutrophils via targeting to SA‐binding immunoglobulin‐like lectin on the surface of neutrophils.^[^ ^32^ ^]^ To investigate the brain penetration capabilities of these nanotheranostics, a brain capillary endothelial cells (BCEC) monolayer transwell system was constructed to simulate in vitro BBB (Figure S24a, Supporting information).^[^ ^33^ ^]^ It was found that all these three nanotheranostics could penetrate across the BCEC monolayer to enter the lower chamber and then be uptaken by C6 cells (Figure S24b, Supporting Information).

**Figure 5:**
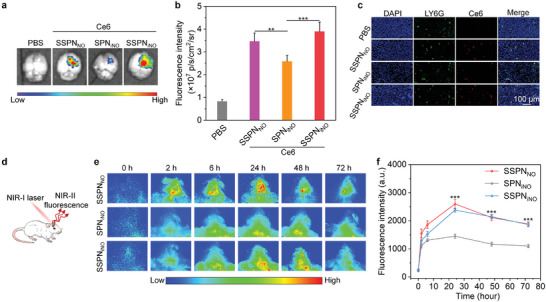
Evaluation of brain targeting effect and NIR‐II fluorescence imaging of orthotopic GBM. a) Fluorescence analysis of brain with tumors after intravenous injection of Ce6‐labeled nanotheranostics for 24 h. b) Analysis of fluorescence intensity of orthotopic GBM sites (*n* = 5, *p* = 0.0017 for SPN_iNO_ vs SSPN_NO,_ ANOVA with Turkey's post‐hoc tests). c) Colocalization analysis of Ce6‐labeled nanotheranostics and neutrophil (LY6G staining) in the tumors of differently treated groups at 24 h post‐injection. d) Scheme of NIR‐II fluorescence imaging of orthotopic GBM using nanotheranostics. e) Continuous in vivo NIR‐II fluorescence imaging of orthotopic GBM after SSPN_NO_, SPN_iNO_, or SSPN_iNO_ injection (GBM‐bearing brains of mice were photographed). f) Analysis of fluorescence intensity in orthotopic GBM after injection of SSPN_NO_, SPN_iNO_, and SSPN_iNO_ at various time points (*n* = 5, unpaired two‐tailed *t* test). Data are presented with mean ± SD (^**^ *p* < 0.01, ^***^ *p* < 0.001, ANOVA with Turkey's post‐hoc tests or unpaired two‐tailed *t* test, for *p* without exact value, *p* < 0.0001).

The in vivo brain targeting capability of nanotheranostics was evaluated using orthotopic GBM model. The brains were extracted from orthotopic GBM‐bearing mice after 24 h systemic administration of Ce6‐labeled nanotheranostics. The brain fluorescence intensities of SSPN_NO_‐ and SSPN_iNO_‐treated mice exhibited significant elevation compared to control and SPN_iNO_‐treated mice (**Figure** **5**a,b). Furthermore, the most prominent fluorescence signals were observed in the tumor sites. The extracted brains were also sectioned into slices for immunofluorescence staining. Although fluorescence signals could also be detected in brain of SPN_iNO_‐treated group, the most intense signals were detected in brain of SSPN_NO_‐ and SSPN_iNO_‐treated groups (Figure 5c). Fluorescence intensity in SSPN_NO_‐ and SSPN_iNO_‐treated groups exhibited an approximately threefold increase compared to that in SPN_iNO_‐treated group (Figure S25, Supporting information). Furthermore, fluorescence signals of Ce6‐labeled SSPN_NO_ and SSPN_iNO_ displayed a high level of colocalization with the staining signals of neutrophils, implying the crucial role of neutrophil hijacking in promoting tumor accumulation of targeting nanotheranostics.^[^ ^34^ ^]^

Imaging‐guided therapy shows great promise for treatments of GBM.^[^ ^35^ ^]^ NIR‐II fluorescence imaging of orthotopic GBM was verified using the nanotheranostics (Figure 5d). After systemic administration, the fluorescence of GBM sites gradually elevated, which similarly reached the peaks at 24 h for SSPN_NO_‐, SPN_iNO_‐ and SSPN_iNO_‐injected mice (Figure 5e). At this time‐point, the signals of GBM sites for SSPN_NO_‐ and SSPN_iNO_‐injected mice were much stronger than those of SPN_iNO_‐injected mice. Quantitative analysis showed that the NIR‐II fluorescence intensities of orthotopic GBM sites for SSPN_NO_‐ and SSPN_iNO_‐injected mice increased by ≈2.0‐fold compared to SPN_iNO_‐injected mice (Figure 5f). This verified the possibility of targeting NIR‐II fluorescence imaging of orthotopic GBM using SSPN_NO_ and SSPN_iNO_. Biodistribution analysis showed that these nanotheranostics mainly accumulated into liver and spleen of living mice (Figure S26, Supporting information). Moreover, fluorescence signals in major organs gradually decreased after injection, which should be due to the effective metabolism of nanotheranostics.

### 2.4 In Vivo Therapeutic Efficacy Evaluation of Orthotopic GBM

**Figure 6:**
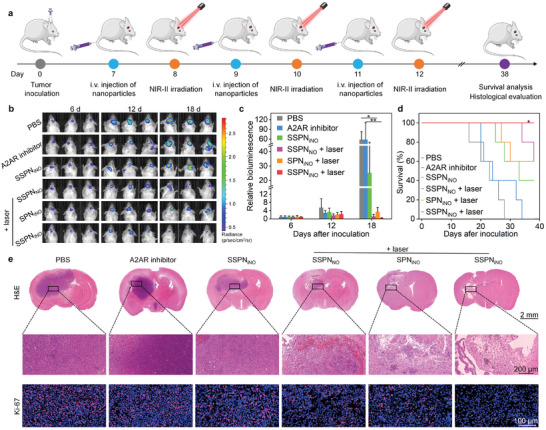
In vivo anti‐GBM effect evaluation. a) Scheme of orthotopic GBM inoculation and treatment protocol of nanotheranostic injection and laser irradiation. b) The bioluminescence images of orthotopic C6‐Luc GBM‐bearing mice after different treatments on Day 6, 12, and 18. c) Bioluminescence intensity analysis of tumors (*n* = 3, *p* = 0.0401 for PBS versus SPN_iNO_ + laser; *p* = 0.0049 for A2AR inhibitor versus SSPN_iNO_ + laser, ANOVA with Turkey's post‐hoc tests). d) Survival curves of orthotopic GBM‐bearing mice for various treatment groups (*n* = 5, *p* = 0.0143, Log‐rank test). e) H&E and Ki‐67 staining images of brain sections from mice with C6 tumors. Data are presented with mean ± SD (^*^ *p* < 0.05, ^**^ *p* < 0.01, ANOVA with Turkey's post‐hoc tests or Log‐rank test).

Orthotopic C6 GBM‐bearing Balb/c mouse models have been used for in vivo antitumor effect evaluation.^[^ ^36^ ^]^ In vivo anti‐tumor efficacy of nanotheranostics was evaluated using orthotopic luciferase (Luc)‐C6 GBM mouse models (**Figure** **6**a). Prior to in vivo evaluation of anti‐GBM efficacy, red blood cells were extracted from mice to test blood safety of nanotheranostics. The hemolysis rates were <5.0% after nanotheranostic incubation even when the concentration reached 50 µg mL^−1^ (Figure S27, Supporting information), suggesting the excellent blood safety of these nanotheranostics. The orthotopic GBM sites were irradiated by NIR‐II laser to mediate in vivo anti‐tumor therapy, and the temperatures of tumors were recorded. The tumor temperatures of SSPN_NO_‐ and SSPN_iNO_‐injected mice were increased by ≈27.0 °C after NIR‐II irradiation for 10 min, 10 °C higher than that of SPN_iNO_‐injected mice under NIR‐II laser exposure (Figure S28, Supporting information). As illustrated in Figure 6b, the injection of SSPN_iNO_ and NIR‐II laser irradiation (10 min) demonstrated a significant tumor growth suppression in comparison to control group, which was evidenced by the notable decrease in the Luc signal intensity in tumor sites. In comparison, SPN_iNO_ + laser and SSPN_NO_ + laser only displayed partial inhibitory effect. The bioluminescence intensity of tumor sites for SPN_iNO_ + laser, SSPN_NO_ + laser and SSPN_iNO_ + laser groups was overall lower than that in PBS, free A2AR inhibitor and SSPN_iNO_ groups (Figure 6c), suggesting the anti‐tumor efficacy of nanotheranostic‐based combinational therapy. SSPN_iNO_ + laser group showed the lowest bioluminescence intensity in tumor sites after 18 days of treatment. The anti‐GBM efficacy was also evaluated by measuring the survival time of mice. In control group, the median survival time of C6 GBM‐bearing models was only 23 days, while free A2AR inhibitor treatment slightly increased the median survival time to 24 days (Figure 6d). In contrast, the survival time was prolonged for SPN_iNO_ + laser, SSPN_NO_ + laser and SSPN_iNO_ + laser groups. Following SPN_iNO_ and SSPN_NO_ administration with NIR‐II laser irradiation, 60% GBM‐bearing mice still survived after tumor inoculation for 38 days. For SSPN_iNO_ + laser group, all GBM‐bearing mice were still alive in the whole observation period. The body weights of mice bearing GBM were also measured to confirm the therapeutic effects. In consistent with previous study,^[^ ^37^ ^]^ body weights of GBM‐bearing mice decreased gradually during tumor growth (Figure S29, Supporting information). The body weight loss were minimal in SSPN_iNO_ + laser group, indicating the best anti‐GBM efficacy.

Histological staining analysis was performed to further study the anti‐GBM efficacy. The tumors could be observed in brain of PBS, free A2AR inhibitor, and SSPN_iNO_ groups, which displayed intact tumor cell morphologies without necrosis due to negligible therapeutic effects (Figure 6e). The tumors were obviously ablated in SPN_iNO_ + laser, SSPN_NO_ + laser, and SSPN_iNO_ + laser groups, and necrosis of tumor cells was detected in these groups. SSPN_iNO_ + laser group showed the highest level of cell necrosis and nearly no tumor cells were observed in brain. Ki‐67 staining indicating the proliferation capability of cancer cells was also performed to evaluate the anti‐GBM efficacy.^[^ ^38^ ^]^ Although Ki‐67 staining signals could be detected in all groups, which were weakest in SSPN_iNO_ + laser group (Figure 6e). The administration of nanotheranostics and NIR‐II laser irradiation led to a marked decrease in Ki‐67 expression, with the signal intensity in SSPN_iNO_ + laser group decreasing to ≈5.0 times lower than that of the control group (Figure S30, Supporting information). Altogether, our results indicated that SSPN_iNO_ could enable the highest anti‐GBM efficacy with NIR‐II laser irradiation. In addition, H&E staining of major organs demonstrated that nanotheranostic‐mediated therapy did not show any damages to heart, lung, liver, kidney and spleen (Figure S31, Supporting information), confirming the good biosafety of this treatment strategy.

### 2.5 Mechanism Studies of Anti‐Tumor Efficacy

**Figure 7:**
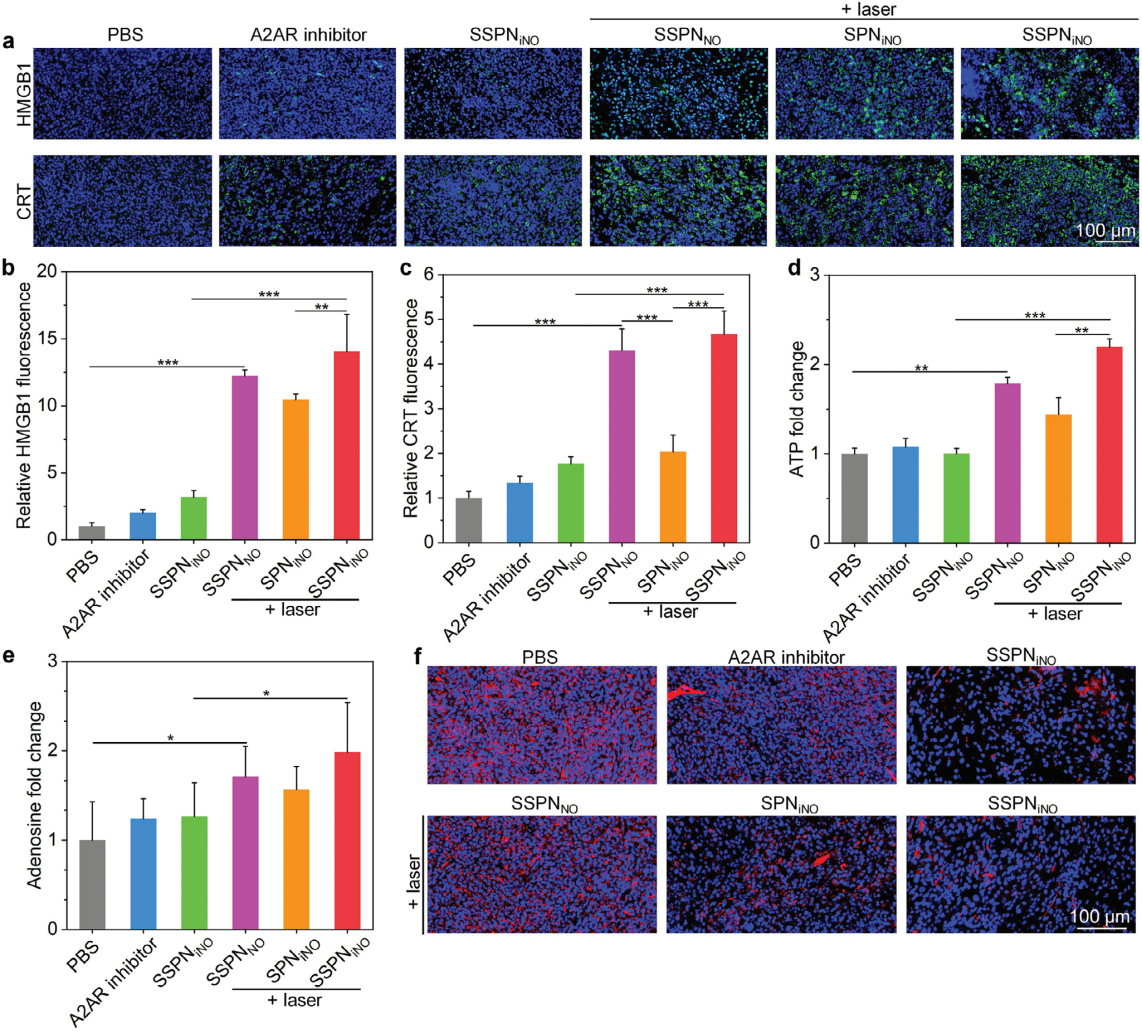
In vivo ICD induction and adenosine metabolism evaluation. a) Immunofluorescence staining images of HMGB1 and CRT in tumor tissues following intravenous injection of PBS, free A2AR inhibitor, SSPN_iNO_, SSPN_NO_, and SPN_iNO_ (200 µL, 300 µg mL^−1^) with or without laser irradiation. b) Analysis of HMGB1 levels in tumors (*n* = 5, *p* = 0.0054 for SPN_iNO_ vs SSPN_iNO_ + laser). c) Analysis of CRT levels in tumors (*n* = 5). d) Relative ATP levels in tumors (*n* = 5, *p* = 0.0024 for PBS vs SSPN_NO_ + laser; *p* = 0.0030 for SPN_iNO_ + laser vs SSPN_iNO_ + laser). e) Relative adenosine levels in tumor tissues (*n* = 5, *p* = 0.0332 for PBS vs SSPN_iNO_ + laser; *p* = 0.0275 for SSPN_iNO_ vs SSPN_iNO_ + laser). f) Immunofluorescence staining images of A2AR in tumors. Data are presented with mean ± SD (^*^ *p* < 0.05, ^**^ *p* < 0.01, ^***^ *p* < 0.001, ANOVA with Turkey's post‐hoc tests, for *p* without exact value, *p* < 0.0001).

To explore the mechanisms of nanotheranostic‐based anti‐tumor efficacy, the ICD effect after treatments were investigated. The green fluorescence signals of HMGB1 and CRT staining were weak in PBS, free A2AR inhibitor, and SSPN_iNO_ groups, which were significantly increased for SPN_iNO_ + laser, SSPN_NO_ + laser, and SSPN_iNO_ + laser groups (**Figure** **7**a). Moreover, HMGB1 and CRT staining signals in SSPN_iNO_ + laser group were stronger than those in SPN_iNO_ + laser and SSPN_NO_ + laser groups. In the SSPN_NO_ + laser and SSPN_iNO_ + laser groups, the HMGB1 fluorescence intensity exhibited respective increases of 1.8‐ and 2.2‐fold compared to the PBS group (Figure 7b). CRT staining signal intensity in SSPN_NO_ + laser and SSPN_iNO_ + laser group was found to increase by 6.1‐ and 7.4‐fold, respectively (Figure 7c). Accordingly, the level of ATP in the tumors was increased by 1.8‐fold for SSPN_NO_ + laser group and 2.2‐fold for SSPN_iNO_ + laser group relative to that in PBS group, respectively (Figure 7d). However, the ATP level did not show significant increase in SSPN_iNO_ and free A2AR inhibitor groups.

ATP could be hydrolyzed to immunosuppressive adenosine by ectonucleotidases in tumor microenvironment, which has been demonstrated to exert immunosuppressive effects by acting on A2AR expressed on various immune cells.^[^ ^39^ ^]^ The level of adenosine in tumors for SSPN_NO_ + laser and SSPN_iNO_ + laser groups increased by ≈1.4‐ and 1.6‐fold as compared to PBS control group, respectively (Figure 7e). However, free A2AR inhibitor and SSPN_iNO_ did not induce obvious increases of adenosine levels in tumor tissues. Immunofluorescence staining was used to verify the modulation of adenosine immunosuppressive tumor microenvironment. The staining signals of A2AR in SPN_iNO_ + laser, SSPN_NO_ + laser and SSPN_iNO_ + laser groups were much weaker than those in PBS and SSPN_iNO_ groups, which were also similar to those in free A2AR inhibitor group (Figure 7f). This revealed the releases of A2AR inhibitor from these nanotheranostics to block the binding of adenosine to A2AR.^[^ ^40^ ^]^ Moreover, SSPN_NO_ + laser and SSPN_iNO_ + laser groups displayed lower A2AR staining signals compared to SPN_iNO_ + laser group (Figure S32, Supporting information). Thus, NIR‐II PTT and NO gas therapy triggered ICD of tumor cells, which could convert the cold GBM into hot phenotype for improving the infiltration of effector T cells into tumor tissues. In addition, A2AR inhibition could reverse the immunosuppressive effect of adenosine for infiltrated T cells, thereby synergistically enhancing the immune response for antitumor efficacy.

**Figure 8:**
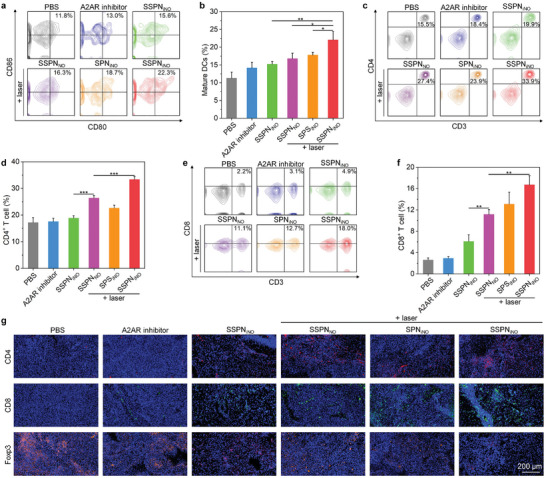
Assessment of anti‐tumor immune response in vivo. a) Percentages of DCs in cervical lymph nodes. b) Analysis of matured DCs in draining cervical lymph nodes (*n* = 3, *p* = 0.0116 for SSPN_NO_ + laser vs SSPN_iNO_ + laser; *p* = 0.0444 for SPN_iNO_ + laser vs SSPN_iNO_ + laser; *p* = 0.0015 for SSPN_iNO_ vs SSPN_iNO_ + laser). c) Percentages of CD3^+^CD4^+^ T cells in spleen of GBM‐bearing mice after various treatments. d) Analysis of CD3^+^CD4^+^ T cells in spleens of mice (*n* = 3, *p* = 0.0002 for SSPN_NO_ + laser vs SSPN_iNO_ + laser). e) Percentages of CD3^+^CD8^+^ T cells in spleens of mice. f) Analysis of CD3^+^CD8^+^ T cells in spleens of mice (*n* = 3, *p* = 0.0052 for SSPN_iNO_ vs SSPN_NO_ + laser; *p* = 0.0025 for SSPN_NO_ + laser vs SSPN_iNO_ + laser). g) Immunofluorescence staining images of tumor sections with CD4, CD8, and Foxp3 staining. Data are presented with mean ± SD (*p < 0.05, **p < 0.01, ***p < 0.001, ANOVA with Turkey's post‐hoc tests, for *p* without exact value, *p* < 0.0001).

To verify the in vivo boosted immune responses, immune cells in spleens, draining lymph nodes, and tumors were investigated. As depicted in **Figure** **8**a, the percentages of DCs in draining lymph nodes exhibited an increase subsequent to various treatment regimes. In SPN_iNO_ + laser, SSPN_NO_ + laser, and SSPN_iNO_ + laser groups, the DCs percentage increased to 17.8%, 16.8%, and 22.1%, respectively (Figure 8b). Then, the CD4^+^ and CD8^+^ T cells in the spleens were also measured. Free A2AR inhibitor and SSPN_iNO_ treatments slightly increased the percentages of CD4^+^ T cells, while obvious increases in CD4^+^ T cell percentages were observed in SPN_iNO_ + laser, SSPN_NO_ + laser, and SSPN_iNO_ + laser groups (Figure 8c). The highest level of CD4^+^ T cells (33.4%) was observed in SSPN_iNO_ + laser group (Figure 8d). Likewise, there was a notable rise in CD8^+^ T cell levels subsequent to the application of nanotheranostics and NIR‐II laser irradiation (Figure 8e,f). It should be noteworthy that the DCs, CD4^+^ T cells, and CD8^+^ T cells were overall higher in SSPN_iNO_ + laser group than those in SSPN_NO_ + laser group, suggesting the modulatory effect of A2AR inhibitor on systemic immune function.^[^ ^40^, ^41^ ^]^

Immunofluorescence CD4, CD8, and Foxp3 staining of orthotopic GBM tumors was also performed to evaluate the local immune response. SPN_iNO_ + laser, SSPN_NO_ + laser, and SSPN_iNO_ + laser groups showed obviously stronger fluorescence signals of CD4 and CD8 compared to PBS, free A2AR inhibitor, and SSPN_iNO_ group (Figure 8g), which indicated the infiltration of more CD4^+^ and CD8^+^ T cells into glioma tissues. Compared to PBS group, the levels of CD4^+^ T cells in tumors increased by 5.7‐, 4.0‐, and 7.7‐fold for SSPN_NO_ + laser, SPN_iNO_ + laser, and SSPN_iNO_ + laser group, respectively (Figure S33, Supporting information). In these groups, the levels of CD8^+^ T cells were also increased by 18.2‐, 21.4‐, and 27.6‐fold in SSPN_NO_ + laser, SPN_iNO_ + laser, and SSPN_iNO_ + laser groups, respectively (Figure S34, Supporting information). The immunosuppressive T_reg_ cells in tumor sites were also evaluated by Foxp3 staining. Foxp3 was highly expressed in GBM tumors as the fluorescence signal was obvious (Figure 8g), which was consistent with previous studies reporting that the tumor microenvironment of GBM was highly immunosuppressive.^[^ ^42^ ^]^ However, the Foxp3 staining signals were weakened in SSPN_NO_ + laser, SPN_iNO_ + laser, and SSPN_iNO_ + laser groups. As compared with the control group, the T_reg_ cells were reduced by 56.0%, 75.0%, and 93.0% for SSPN_NO_ + laser, SSPN_iNO_ + laser, and SSPN_iNO_ + laser, respectively (Figure S35, Supporting information). These results demonstrated the highest CD4^+^ and CD8^+^ T cells, but the lowest T_reg_ cells in orthotopic GBM tumors of SSPN_iNO_ + laser group, indicating the strongest immunological effect. Combination of NIR‐II PTT and NO gas therapy induced an amplified ICD effect for activating the immune response, which synergized with A2AR inhibitor to achieve the immune response potentiation.

## 3 Conclusion

Herein, we have constructed neutrophil‐targeting nanotheranostics (SSPN_iNO_) that contained two SPs, a thermal‐responsive NO donor and A2AR inhibitor for NIR‐II fluorescence imaging‐guided combination therapy of orthotopic GBM. The surface of SSPN_iNO_ was embellished with a neutrophil‐targeting ligand, which enabled their adherence to neutrophils to cross BBB and achieve effective delivery into orthotopic GBM sites. Dopings of two SPs enabled NIR‐II fluorescence imaging and PTT. Upon NIR‐II laser irradiation, SSPN_iNO_ could convert photon into heat, leading to increased local temperature and NO release from thermal‐responsive NO donor. Thus, in situ NO release and photothermal effect could be remotely triggered by NIR‐II laser after systemic injection of SSPN_iNO_ into the orthotopic GBM‐bearing mice, which induced ICD effect and shift of immunologically “cold” tumors to “hot” ones. Additionally, SSPN_iNO_ delivered A2AR inhibitor to block the inhibition of adenosine pathway, further boosting immunological effect to eradicate orthotopic GBM. To our best knowledge, this is the first investigation on the pioneering application of polymer nanotheranostic for NIR‐II fluorescence imaging‐guided PTT‐NO‐immunotherapy of orthotopic GBM.

Given the good tissue penetration capability of NIR‐II light, this nanotheranostic design is expected to be suitable for treatments of other orthotopic tumors, providing good opportunities for clinical translation. Although these promising results, the nanotheranostics still have some challenges that restrict their translation into clinical practices. More efforts should be made for enhancing degradability and metabolism to ensure excellent in vivo biosafety. The components and targeting ability of nanotheranostics can be further optimized to improve scalability for the applications in different types of tumors and other nervous system diseases. In addition, clinical regulatory hurdles of these nanotheranostics should be considered to increase the possibility of their translation.

## 4 Experimental Section

### Synthesis of DSPE‐PEG‐GSNO and DSPE‐PEG‐SA

DSPE‐PEG‐NHS and GSNO were dissolved in anhydrous DMSO at a molar ratio of 1:3. The reaction solution was protected from light and kept stirring for 72 h. Afterward, the reaction products were purified by dialysis. Finally, DSPE‐PEG‐GSNO was obtained via lyophilization. Similarly, DSPE‐PEG‐SA was synthesized by first activating the carboxyl group of SA using EDC/NHS mixture (SA:EDC:NHS, 1:5:5, mol/mol/mol) for 2 h. Afterward, DSPE‐PEG‐NH_2_ was added (DSPE‐PEG‐NH_2_:SA, 1:5) and stirred for additional 72 h. The final DSPE‐PEG‐SA was purified and freeze‐dried.

### Preparation of SP‐Based Nanotheranostics

SP1 (0.5 mg), SP2 (0.2 mg), A2AR inhibitor (1.0 mg), DSPE‐PEG‐SA (5 mg) and DSPE‐PEG‐GSNO (20 mg) were dissolved in 1 mL tetrahydrofuran (THF) and the mixed solution was injected into THF/phosphate buffer saline (PBS) (10 mL, v/v = 1:9) solution to prepare SA‐modified SP‐based nanotheranostics (SSPN_iNO_). After sonication for 20 min, THF was evaporated overnight under N_2_ atmosphere. The nanoparticle solutions were filtered and subsequently stored at 4 °C in the dark. The solutions were concentrated by ultrafiltration (30 kDa). The blank SA‐modified nanoparticles (SSPN_NO_) and A2AR inhibitor‐loaded unmodified nanoparticles (SPN_iNO_) were also prepared following similar procedures. Ce6 (0.2 mg) was added during preparation to construct Ce6‐labeled nanotheranostics. The concentrations of A2AR inhibitor were determined by HPLC. The drug loading and entrapment efficiencies of A2AR inhibitor were determined as previously described.^[^ ^41^ ^]^

### In Vitro Photothermal Performance of Nanotheranostics

Nanoparticles (SSPN_NO_, SPN_iNO_, and SSPN_iNO_) in PBS solutions at concentrations ranging from 12.5 to 100 µg mL^−1^ were exposed to NIR‐II laser (1064 nm) irradiation for 6 min at varying power densities, and the temperature changes were recorded. The photothermal conversion efficiency (η) was calculated as described previously.^[^ ^43^ ^]^

### Analysis of NO Generation

The generation of NO by nanotheranostic in aqueous solutions was measured with Griess kit.^[^ ^16^, ^25^ ^]^ SSPN_iNO_ were mixed with Griess solution before exposing to NIR‐II laser irradiation (1.0 W cm^−2^), and the absorbance changes (540 nm) were dynamically monitored. Additionally, a solution of SSPN_iNO_ (50 µg mL^−1^) was mixed with fluorescence DAR‐1 probe (20 µm) and exposed to laser. The fluorescence intensities before and after irradiation were recorded by fluorescence spectrophotometer.

### In Vitro Release of A2AR Inhibitor

The release of A2AR inhibitor from SSPN_iNO_ was investigated by dialysis method. The SSPN_iNO_ solution (A2AR inhibitor, 125 µg mL^−1^, 1 mL) was placed into dialysis bag (Molecular weight cut‐off, 1000 Da) and then submerged in release medium. Samples were withdrawn at the predetermined time points. The concentrations of the A2AR inhibitor in the solutions was analyzed using HPLC.^[^ ^41^ ^]^

### In Vitro Assessment of Cytotoxicity and Therapeutic Efficacy

The cytotoxicity of the as‐prepared nanotheranostics on C6‐Luc cells was evaluated using CCK‐8 assay. Cells were seeded (10^4^/well) and cultured for 24 h. Then, cells were treated with varying concentrations of SSPN_NO_, SPN_iNO_, or SSPN_iNO_ for another 24 h. For in vitro assessment of therapeutic efficacy, C6‐Luc cells were treated with PBS, A2AR inhibtor, SSPN_NO_, SPN_iNO_, or SSPN_iNO_ (50 µg mL^−1^). NIR‐II irradiation (1.0 W cm^−2^, 5 min) was applied after cells were treated with SSPN_NO_, SPN_iNO_, or SSPN_iNO_ for 12 h. After re‐incubation for another 12 h, CCK‐8 assay was applied to evaluate cell viability. C6 cells were treated with PBS, A2AR inhibitor (10 µg mL^−1^) or nanotheranostics (50 µg mL^−1^) for 4 h, then subjected to NIR‐II laser irradiation (1.0 W cm^−2^, 5 min). After a further 4 h of incubation, the stained cells were observed using Leica fluorescence microscope.

### Detection of NO Generation in Cells

The C6 cells were treated with SSPN_NO_, SPN_iNO_, or SSPN_iNO_ (50 µg mL^−1^) for 12 h and then incubated with DMEM (5 µM DAF‐FM DA) for 30 min. Following 5 min of NIR‐II laser irradiation (1.0 W cm^−2^), the cells were washed with PBS and intracellular NO was assessed using fluorescence microscope.

### Detection of ICD and ΔΨ_m_ In Vitro

For HMGB1 detection, C6 cells were inoculated into 24‐well plate and treated with PBS, A2AR inhibitor, SSPN_NO_, SPN_iNO_ or SSPN_iNO_ with or without NIR‐II laser irradiation. After various treatments, the cell culture medium from each well was collected, and the supernatants were obtained via centrifugation. The concentrations of HMGB1 in the supernatants were quantified using ELISA kit (JYM0485Mo, ColorfulGene Biological Technology Co., LTD, Wuhan, China). For CRT detection, the treated C6 cells were incubated with CRT antibodies (1:100) for 30 min, followed by second antibodies (1:400) labeled with Alexa Fluor 488. Then the fluorescence signal of CRT staining was observed by fluorescence microscope. ATP assay kit (Beyotime Biotechnology) was utilized to measure the ATP level of cell culture supernatants. The adenosine levels of cells after various treatments were also measured by ELISA kit. C6‐Luc cells were treated with PBS, A2AR inhibitor, SSPN_NO_, SPN_iNO_, or SSPN_iNO_ (50 µg mL^−1^), and irradiated by NIR‐II laser (5 min). Following treatment, cells were incubated with the JC‐1 fluorescent probe, and subsequent analysis was conducted via flow cytometry to quantify the ratio of JC‐1 monomers to aggregates.^[^ ^44^ ^]^

### Cellular Uptake of Nanotheranostics in Distinct Cells

To evaluate the cellular uptake of nanotheranostics by different cells, 25 µg mL^−1^ of Ce6‐labeled nanotheranostics were employed to incubate with C6 cells, RAW264.7 macrophages, and neutrophils. Nanotheranostics were incubated with C6 and RAW cells after 24 h of seeding and culture. After 3 h of co‐incubation, the cells were collected for flow cytometry analysis. Neutrophils were isolated using Percoll gradient centrifugation.^[^ ^45^ ^]^ Briefly, bone marrows from the femur and tibia of Balb/c mice were flushed with RPMI‐1640 medium. The cell suspension was centrifuged and then resuspended. This suspension was layered onto a Percoll density gradient (Cytiva, Uppsala, Sweden) composed of 55%, 65%, and 78% (v/v) Percoll in PBS. Following centrifugation at 350 g for 30 min, neutrophils were harvested, washed, and finally resuspended in fresh medium. The uptake of nanotheranostics by neutrophils was evaluated.

### In Vitro Evaluation of BBB Penetrating Capability of Nanotheranostics

To assess the BBB penetration capability of nanotheranostics, the in vitro BBB model was constructed following previous protocols.^[^ ^33^ ^]^ In brief, bEnd.3 cells were seeded on the upper chambers of six‐well transwell plates and incubated for 7 days with regular medium replacement. The BBB model establishment was confirmed upon reaching a trans‐endothelial electrical resistance value exceeding 200 Ω cm^2^. Then, C6 cells were cultured on the lower chambers of transwell plates. After attachment for 24 h, Ce6‐labeled nanotheranostics dispersed in fresh culture medium (50 µg mL^−1^) were added into the upper chambers. Following 24 h of incubation, the C6 cells were observed using confocal fluorescence microscope. DAPI was used to stain cell nuclei.

### Establishment of Orthotopic GBM Models

Female Balb/c mice (≈20 g) were obtained from JieSiJie Laboratory Animal Co., Ltd (Shanghai, China). Animal studies were conducted in accordance with the approved procedures by the Animal Care and Use Committee of Donghua University (approve number: DHUEC‐STCSM‐2021‐24). To establish orthotopic GBM models, 2 × 10^6^ C6‐Luc cells were intracerebrally injected into the right striatum of mice.

### In Vivo Assessment of Tumor Accumulation of Nanotheranostics

To investigate the brain‐ and tumor‐targeting ability of nanotheranostics in vivo, orthotopic C6‐bearing Balb/c mice were used. After intravenous injection of different Ce6‐labeled nanotheranostics for 24 h, the mouse brains were extracted for IVIS imaging (Caliper PerkinElmer, Waltham, MA, USA) with a fluorescence filter set (Ex/Em = 640/710 nm). The Ce6 fluorescence intensity of brain was quantified based on the regions of interest. Immunofluorescence staining of brain tumor slices was conducted to evaluate the colocalization of nanotheranostics with neutrophils using FITC‐conjugated anti‐Ly‐6G antibody (1:100).

### In Vivo NIR‐II Fluorescence Imaging of Orthotopic GBM

NIR‐II fluorescence imaging capability of nanotheranostics was assessed by injecting nanoparticles intravenously into C6‐bearing mice. Then NIR‐II fluorescence images of tumor sites in GBM models were captured intermittently using a V‐NIR‐II imaging system (Digi‐United Biotech, Shanghai, China).

### In Vivo Evaluation of Anti‐GBM Efficacy

The C6‐Luc GBM‐bearing Balb/c mice were injected with PBS, A2AR inhibitor (1 mg kg^−1^), SSPN_NO_, SPN_iNO_ or SSPN_iNO_ (300 µg mL^−1^) via tail vein (200 µL/mouse). 24 h after injection, the tumor sites were irradiated with 1064 nm laser (1.0 W cm^−2^) for 10 min. The administration of nanoparticles and laser irradiation was performed on alternate days for a total of three times. In vivo orthotopic GBM growths were monitored using IVIS system after intraperitoneal injection of luciferin substrate (100 mg kg^−1^). In vivo survival analysis was conducted on mice bearing orthotopic C6 tumors. To evaluate cell proliferation and apoptosis/necrosis of tumors, brains were extracted from the C6‐Luc‐bearing mice 7 days after different treatments, and cut into sections for Ki‐67 and hematoxylin and eosin (H&E) staining.

### Assessment of ICD and Adenosine Levels

Orthotopic C6‐Luc‐bearing mice were intravenously administered with PBS, A2AR inhibitor, SSPN_NO_, SPN_iNO_, and SSPN_iNO_, and the tumors were irradiated by 1064‐nm laser (1.0 W cm^−2^, 10 min). The mice were euthanized and tumors were harvested for ATP and adenosine measurements using ATP assay kit and ELISA kit, respectively. For HMGB1 and CRT assessment, immunofluorescence staining of brain slices from C6‐Luc‐bearing mice was conducted. Slices were stained with anti‐CRT (1:200) or anti‐HMGB1 (1:500) primary antibody and FITC‐conjugated secondary antibody (1:200). Fluorescence signals of the stained slices were visualized using fluorescence microscope.

### Assessment of Matured DCs in Lymph Nodes

Seven days after various treatments, tumor‐draining cervical lymph nodes were harvested from C6‐Luc‐bearing mice and subsequently homogenized in PBS solution. A single cell suspension was obtained through filtration using 70‐µm strainers, followed by staining of cells with antibodies. After removing the unbound antibodies, flow cytometry was utilized for cell analysis.

### Assessment of T cells in Spleen

The treated mice were sacrificed to extract the spleen after 7 days of treatments. Spleen‐derived single cell suspensions were prepared by mechanical disruption and subsequent filtration. Lymphocytes were isolated from the suspension using lymphocyte separation medium. Subsequently, these lymphocytes were stained with antibodies to characterize T cell populations. DAPI staining was performed to exclude dead cells. Flow cytometry was used to analyze the stained cells after removing the excess antibodies.

### Assessment of A2AR Expression and T Cell Levels in GBM

To assess the A2AR expression and T cell levels in orthotopic GBM, brain slices from C6‐Luc‐bearing mice were used for immunofluorescence staining.

### Statistical Analysis

Pre‐processing of data was not involved in this study. Data presentation were expressed as mean ± standard deviation (SD). The sample size (n) was provided for each statistical analysis. Analysis of significant differences was conducted using two‐tailed Student's *t*‐tests and one‐way analysis of variance (ANOVA) with Tukey's post‐hoc tests. The significant differences of *p* < 0.05, < 0.01, and < 0.001 were marked with *, **, and ***, respectively. The Software Graphpad 8.0 was used for statistical analysis.

## Conflict of Interest

The authors declare no conflict of interest.

## Supporting information

Supporting Information

## Data Availability

The data that support the findings of this study are available from the corresponding author upon reasonable request.

## References

[1] a) H. Athanassiou , M. Synodinou , E. Maragoudakis , M. Paraskevaidis , C. Verigos , D. Misailidou , D. Antonadou , G. Saris , K. Beroukas , P. Karageorgis , J. Clin. Oncol. 2005, 23, 2372; 15800329 10.1200/JCO.2005.00.331

[2] A. C. Tan , D. M. Ashley , G. Y. Lopez , M. Malinzak , H. S. Friedman , M. Khasraw , CA Cancer J. Clin. 2020, 70, 299. 32478924 10.3322/caac.21613

[3] J. H. Sampson , M. D. Gunn , P. E. Fecci , D. M. Ashley , Nat. Rev. Cancer 2020, 20, 12. 31806885 10.1038/s41568-019-0224-7 PMC7327710

[4] a) C. M. Jackson , J. Choi , M. Lim , Nat. Immunol. 2019, 20, 1100; 31358997 10.1038/s41590-019-0433-y

[5] a) T. F. Gajewski , H. Schreiber , Y. X. Fu , Nat. Immunol. 2013, 14, 1014; 24048123 10.1038/ni.2703 PMC4118725

[6] J. Zhang , C. Chen , A. Li , W. Jing , P. Sun , X. Huang , Y. Liu , S. Zhang , W. Du , R. Zhang , Y. Liu , A. Gong , J. Wu , X. Jiang , Nat. Nanotechnol. 2021, 16, 538. 33526838 10.1038/s41565-020-00843-7

[7] A. van Weverwijk , K. E. d. Visser , Nat. Rev. Cancer 2023, 23, 193. 36717668 10.1038/s41568-022-00544-4

[8] D. V. Krysko , A. D. Garg , A. Kaczmarek , O. Krysko , P. Agostinis , P. Vandenabeele , Nat. Rev. Cancer 2012, 12, 860. 23151605 10.1038/nrc3380

[9] J. Chen , C. Ning , Z. Zhou , P. Yu , Y. Zhu , G. Tan , C. Mao , Prog. Mater. Sci. 2019, 99, 1. 30568319 10.1016/j.pmatsci.2018.07.005 PMC6295417

[10] a) B. Guo , Z. H. Sheng , D. H. Hu , C. B. Liu , H. R. Zheng , B. Liu , Adv. Mater. 2018, 30, 1802591; 10.1002/adma.201802591 30129690

[11] a) J. Li , D. Cui , J. Huang , S. He , Z. Yang , Y. Zhang , Y. Luo , K. Pu , Angew. Chem., Int. Ed. 2019, 58, 12680; 10.1002/anie.201906288 31278823

[12] a) E. E. Sweeney , J. Cano‐Mejia , R. Fernandes , Small 2018, 14, 1800678; 10.1002/smll.201800678 29665282

[13] a) H. S. Hao , M. Yu , Y. F. Yi , S. J. Sun , X. Y. Huang , C. Y. Huang , Y. Q. Liu , W. X. Huang , J. Q. Wang , J. Zhao , M. Y. Wu , Chem. Eng. J. 2022, 437, 135371;

[14] A. Garcia‐Ortiz , J. M. Serrador , Trends Mol. Med. 2018, 24, 412. 29519621 10.1016/j.molmed.2018.02.002

[15] Y. C. Sung , P. R. Jin , L. A. Chu , F. F. Hsu , M. R. Wang , C. C. Chang , S. J. Chiou , J. T. Qiu , D. Y. Gao , C. C. Lin , Y. S. Chen , Y. C. Hsu , J. Wang , F. N. Wang , P. L. Yu , A. S. Chiang , A. Y. Wu , J. J. Ko , C. P. Lai , T. T. Lu , Y. Chen , Nat. Nanotechnol. 2019, 14, 1160. 31740794 10.1038/s41565-019-0570-3

[16] W. Jiang , W. Dong , M. Li , Z. Guo , Q. Wang , Y. Liu , Y. Bi , H. Zhou , Y. Wang , ACS Nano 2022, 16, 3881. 35238549 10.1021/acsnano.1c09048

[17] J. Kim , S. N. Thomas , Pharmacol. Rev. 2022, 74, 1146. 36180108 10.1124/pharmrev.121.000500 PMC9553106

[18] a) J. Li , R. Jiang , Q. Wang , X. Li , X. Hu , Y. Yuan , X. Lu , W. Wang , W. Huang , Q. Fan , Biomaterials 2019, 217, 119304; 31279099 10.1016/j.biomaterials.2019.119304

[19] a) Y. S. Liu , Y. Li , S. Koo , Y. Sun , Y. X. Liu , X. Liu , Y. N. Pan , Z. Y. Zhang , M. X. Du , S. Y. Lu , X. Qiao , J. F. Gao , X. B. Wang , Z. X. Deng , X. L. Meng , Y. L. Xiao , J. S. Kim , X. C. Hong , Chem. Rev. 2022, 122, 209; 34664951 10.1021/acs.chemrev.1c00553

[20] a) H. S. Kim , M. J. Goh , N. Kim , C. G. Choi , S. J. Kim , J. H. Kim , Radiology 2014, 273, 831; 24885857 10.1148/radiol.14132868

[21] X. L. Cai , A. Bandla , C. K. Chuan , G. Magarajah , L. D. Liao , D. B. L. Teh , B. K. Kennedy , N. V. Thakor , B. Liu , Mater. Horiz. 2019, 6, 311.

[22] F. Acerbi , M. Broggi , K. M. Schebesch , J. Hohne , C. Cavallo , C. De Laurentis , M. Eoli , E. Anghileri , M. Servida , C. Boffano , B. Pollo , M. Schiariti , S. Visintini , C. Montomoli , L. Bosio , E. L. a. Corte , G. Broggi , A. Brawanski , P. Ferroli , Clin. Cancer Res. 2018, 24, 52. 29018053 10.1158/1078-0432.CCR-17-1184

[23] a) R. An , L. Liu , S. Wei , Z. Huang , L. Qiu , J. Lin , H. Liu , D. Ye , ACS Nano 2022, 16, 20607; 36508254 10.1021/acsnano.2c07491

[24] J. Xue , Z. Zhao , L. Zhang , L. Xue , S. Shen , Y. Wen , Z. Wei , L. Wang , L. Kong , H. Sun , Nat. Nanotechnol. 2017, 12, 692. 28650441 10.1038/nnano.2017.54

[25] J. Liu , J. Li , Y. Shi , A. Zhu , M. Ding , N. Yu , Y. Liu , P. Wang , J. Li , Y. Liu , Small Struct. 2024, 5, 2300508.

[26] R. R. Guo , Y. Tian , Y. J. Wang , W. L. Yang , Adv. Funct. Mater. 2017, 27, 1606398.

[27] W. Wu , M. Chen , T. Luo , Y. Fan , J. Zhang , Y. Zhang , Q. Zhang , A. Sapin‐Minet , C. Gaucher , X. Xia , Acta Biomater. 2020, 103, 259. 31846803 10.1016/j.actbio.2019.12.016

[28] A. Banstola , K. Poudel , J. O. Kim , J. H. Jeong , S. Yook , J. Controlled Release 2021, 337, 505. 10.1016/j.jconrel.2021.07.038 34314800

[29] a) L. Zhou , P. Zhang , H. Wang , D. Wang , Y. Li , Acc. Chem. Res. 2020, 53, 1761; 32819102 10.1021/acs.accounts.0c00254

[30] E. Giampazolias , B. Zunino , S. Dhayade , F. Bock , C. Cloix , K. Cao , A. Roca , J. Lopez , G. Ichim , E. Proics , C. Rubio‐Patino , L. Fort , N. Yatim , E. Woodham , S. Orozco , L. Taraborrelli , N. Peltzer , D. Lecis , L. Machesky , H. Walczak , M. L. Albert , S. Milling , A. Oberst , J. E. Ricci , K. M. Ryan , K. Blyth , S. W. G. Tait , Nat. Cell Biol. 2017, 19, 1116. 28846096 10.1038/ncb3596 PMC5624512

[31] B. Abadi , N. Yazdanpanah , A. Nokhodchi , N. Rezaei , Adv. Drug Delivery Rev. 2021, 179, 114035. 10.1016/j.addr.2021.114035 34740765

[32] a) S. Wang , X. Lai , C. Li , M. Chen , M. Hu , X. Liu , Y. Song , Y. Deng , J. Controlled Release 2021, 337, 612; 10.1016/j.jconrel.2021.07.044 34332025

[33] Z. Zhou , K. Li , Y. Guo , P. Liu , Q. Chen , H. Fan , T. Sun , C. Jiang , ACS Nano 2023, 17, 7847. 37039779 10.1021/acsnano.3c01140

[34] Q. Qiu , C. Li , X. Yan , H. Zhang , X. Luo , X. Gao , X. Liu , Y. Song , Y. Deng , Biomaterials 2021, 269, 120652. 33450581 10.1016/j.biomaterials.2021.120652

[35] S. Hu , H. M. Kang , Y. Baek , G. El Fakhri , A. R. Kuang , H. S. Choi , Adv. Healthcare Mater. 2018, 7, 1800066. 10.1002/adhm.201800066 PMC6105507 29719137

[36] a) M. Ding , A. Zhu , Y. Zhang , J. Liu , L. Lin , X. Wang , J. Li , Nano Today 2024, 57, 102398;

[37] S. Zhou , Y. Huang , Y. Chen , Y. Liu , L. Xie , Y. You , S. Tong , J. Xu , G. Jiang , Q. Song , N. Mei , F. Ma , X. Gao , H. Chen , J. Chen , Nat. Commun. 2023, 14, 435. 36702831 10.1038/s41467-023-35957-8 PMC9880004

[38] Y. J. Liu , D. Y. Zhang , Y. An , Y. J. Sun , J. Li , M. Zheng , Y. Zou , B. Y. Shi , Nano Today 2023, 49, 101790.

[39] A. K. Moesta , X. Y. Li , M. J. Smyth , Nat. Rev. Immunol. 2020, 20, 739. 32728220 10.1038/s41577-020-0376-4

[40] Y. Liu , Y. Liu , D. Xu , J. Zang , X. Zheng , Y. Zhao , Y. Li , R. He , S. Ruan , H. Dong , J. Gu , Y. Yang , Q. Cheng , Y. Li , Adv. Sci. 2022, 9, 2104182. 10.1002/advs.202104182 PMC9108638 35306759

[41] N. Y. Yu , M. Li , Y. J. Zhang , F. S. Wang , X. R. Yu , R. Cai , J. C. Li , Nano Today 2023, 52, 101944.

[42] a) D. H. Heiland , V. M. Ravi , S. P. Behringer , J. H. Frenking , J. Wurm , K. Joseph , N. W. C. Garrelfs , J. Strahle , S. Heynckes , J. Grauvogel , P. Franco , I. Mader , M. Schneider , A. L. Potthoff , D. Delev , U. G. Hofmann , C. Fung , J. Beck , R. Sankowski , M. Prinz , O. Schnell , Nat. Commun. 2019, 10, 2541; 31186414 10.1038/s41467-019-10493-6 PMC6559986

[43] C. Xu , Y. Jiang , Y. Han , K. Pu , R. Zhang , Adv. Mater. 2021, 33, 2008061. 10.1002/adma.202008061 33634897

[44] M. Li , Y. Liu , Y. J. Zhang , N. Y. Yu , J. C. Li , Adv. Sci. 2023, 10, 2305150. 10.1002/advs.202305150 PMC10724419 37870196

[45] S. Li , M. Li , S. Huo , Q. Wang , J. Chen , S. Ding , Z. Zeng , W. Zhou , Y. Wang , J. Wang , Adv. Mater. 2021, 33, 2006160. 10.1002/adma.202006160 33296121

