# Supplementary material for: Neutrophil‐Targeting Semiconducting Polymer Nanotheranostics for NIR‐II Fluorescence Imaging‐Guided Photothermal‐NO‐Immunotherapy of Orthotopic Glioblastoma
Source: Adv Sci (Weinh). 2024 Aug 19;11(39):2406750. doi: 10.1002/advs.202406750 (PMC11497063; doi:10.1002/advs.202406750)
Supplement: Supplementary file 1 — Supporting Information [file ADVS-11-2406750-s001.docx]

Copyright WILEY-VCH Verlag GmbH & Co. KGaA, 69469 Weinheim, Germany, 2018.

Supporting Information

Neutrophil-Targeting Semiconducting Polymer Nanotheranostics for NIR-II Fluorescence Imaging-Guided Photothermal-NO-Immunotherapy of Orthotopic Glioblastoma

Jiansheng Liu, Danling Cheng, Anni Zhu, Mengbin Ding, Ningyue Yu, and Jingchao Li*

Dr. J. Liu, D. Cheng, A. Zhu, M. Ding, Ni. Yu, Prof. J. Li

State Key Laboratory for Modification of Chemical Fibers and Polymer Materials, College of Biological Science and Medical Engineering, Donghua University, Shanghai 201620, China

E-mail: jcli@dhu.edu.cn

# 1. Supporting Figures


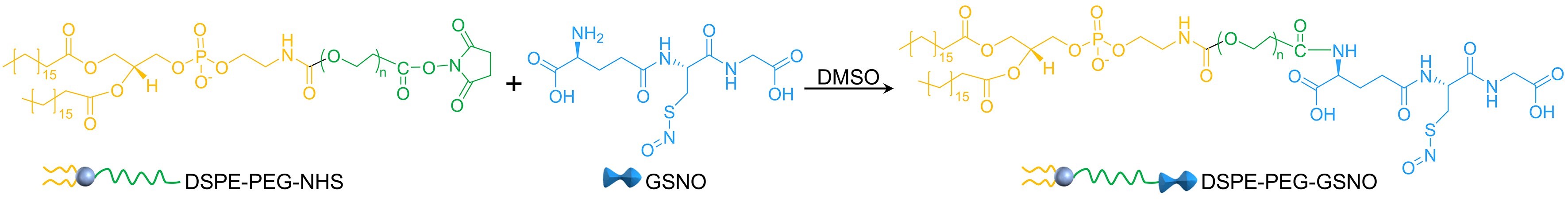


**Figure S1.** Chemical synthesis route of DSPE-PEG-GSNO.


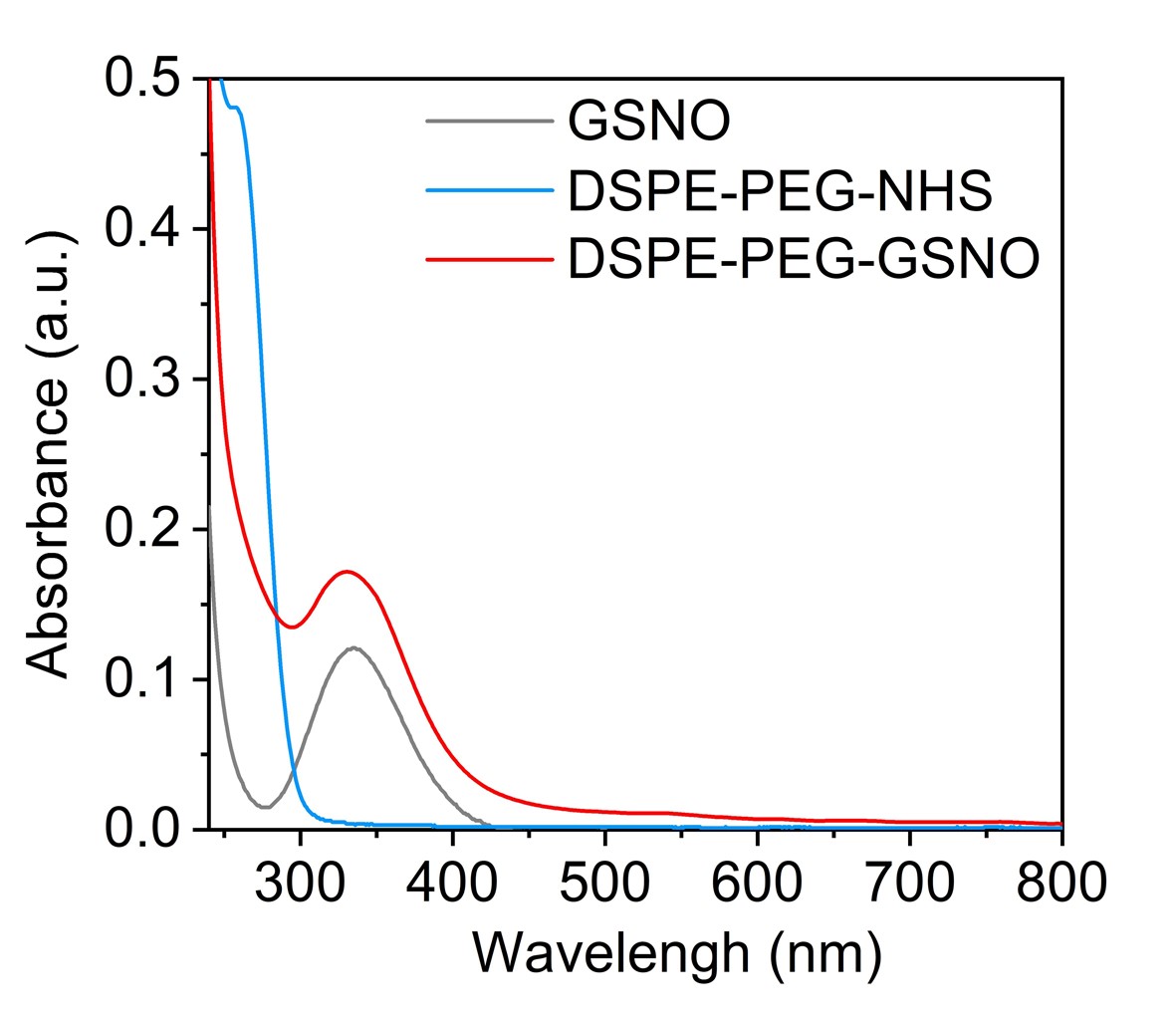


**Figure S2.** UV-vis spectra of GSNO, DSPE-PEG-NHS and DSPE-PEG-GSNO.


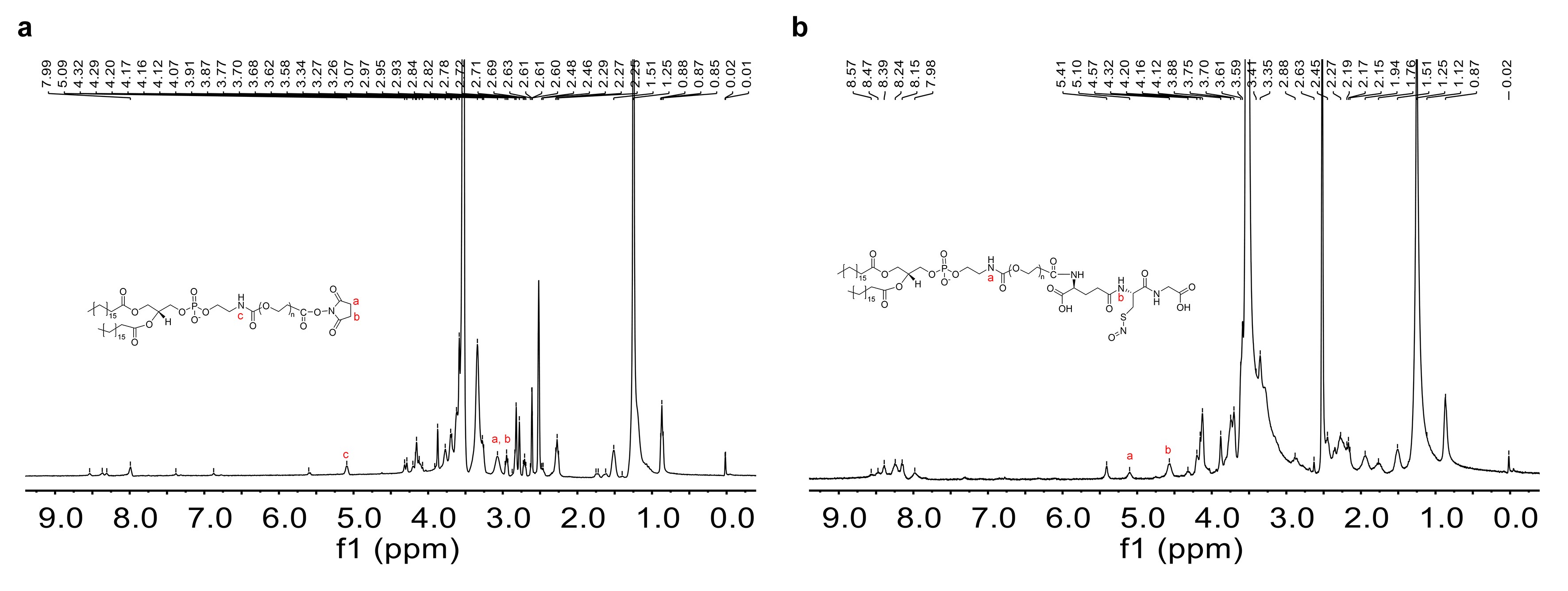


**Figure S3.** ^1^H NMR spectra (400 MHz, DMSO-*d_6_*, δ, ppm) of (a) DSPE-PEG-NHS and (b) DSPE-PEG-GSNO.


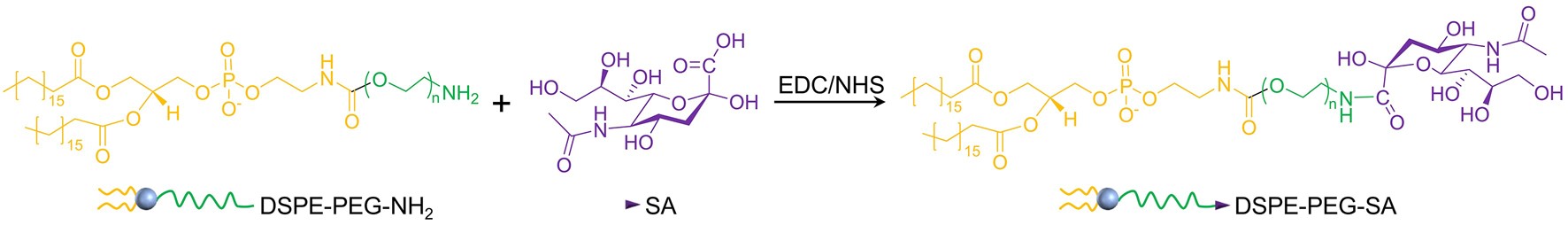


**Figure S4.** Synthesis route of DSPE-PEG-SA.


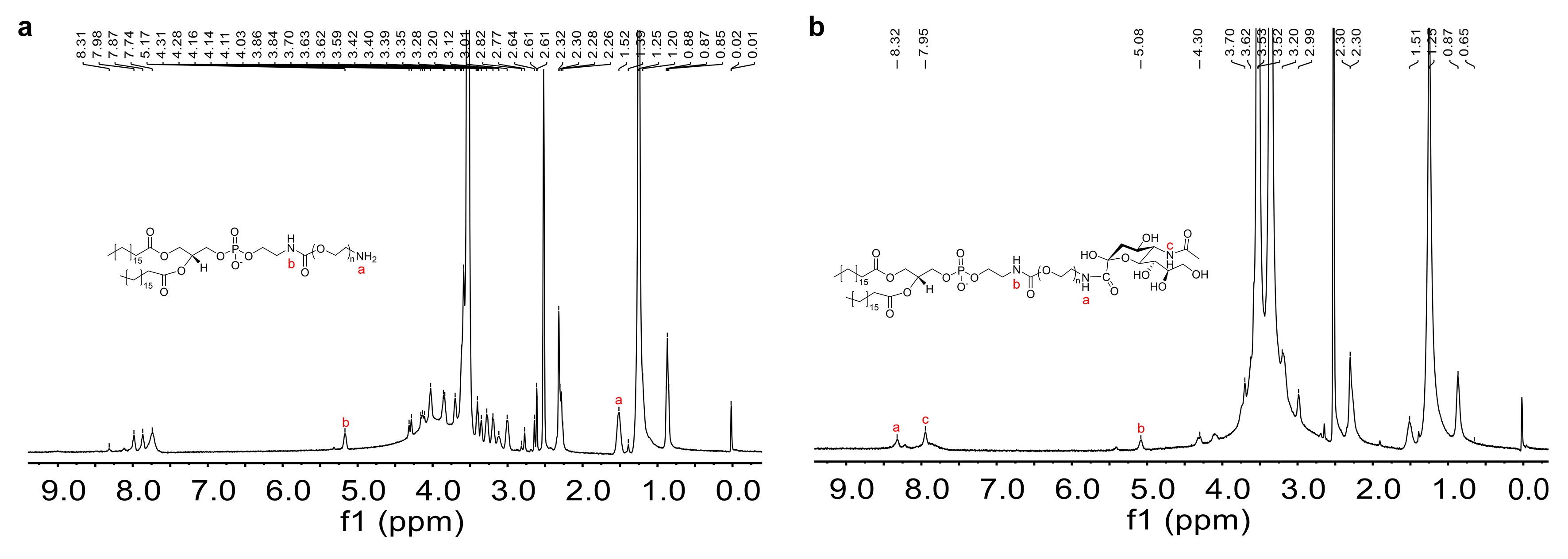


**Figure S5.** ^1^H NMR spectra (400 MHz, DMSO-*d_6_*, δ, ppm) of (a) DSPE-PEG-NH_2_ and (b) DSPE-PEG-SA.


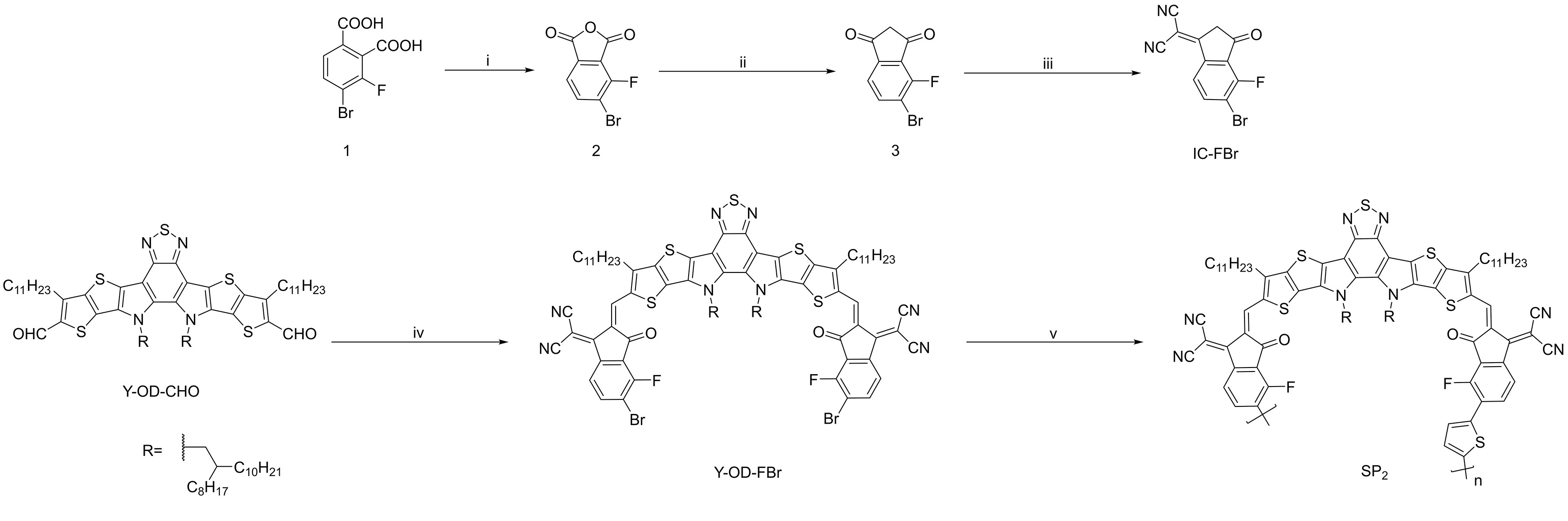


**Figure S6.** Synthesis routes of SP2. i) Acetic anhydride (Ac_2_O), 140 °C, 3 h; ii) Tert-butyl acetoacetate, Ac_2_O, triethylamine, 65 °C, 12 h; iii) Malononitrile, sodium acetate, EtOH, room temperature, 24 h; iv) Pyridine, CHCl_3_, 65 °C, 30 min; v) Distannylated monomer thiophene-tin, Pd_2_(dba)_3_, P(o-tol)_3_, toluene, 110 °C, 72 h.

**
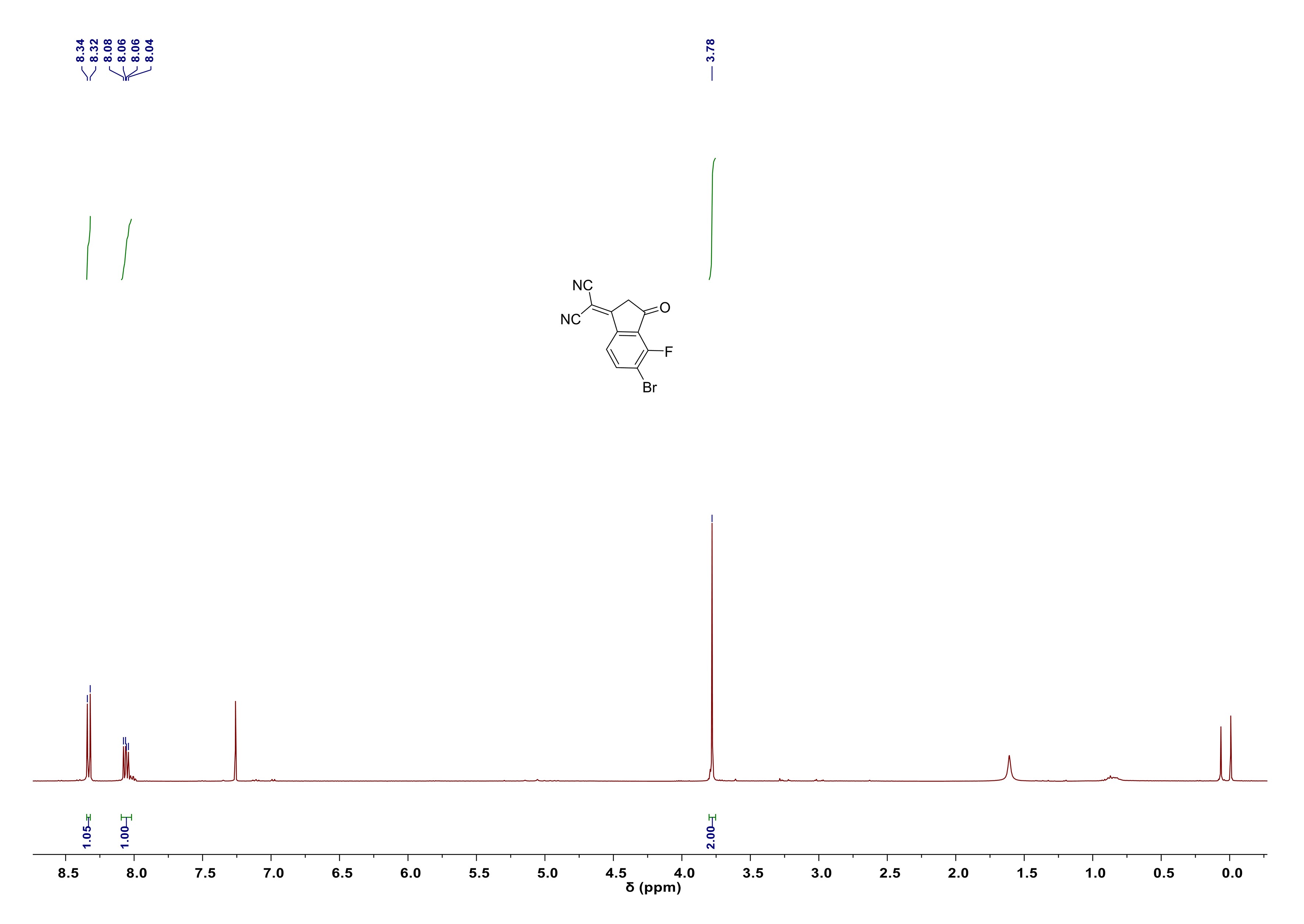
**

**Figure S7.** ^1^H NMR spectrum of **IC-FBr** (400 MHz, CDCl_3_): *δ* 8.33 (d, *J* = 8.4 Hz, 1H), 8.06 (dd, *J* = 8.5, 6.1 Hz, 1H), 3.78 (s, 2H).

**
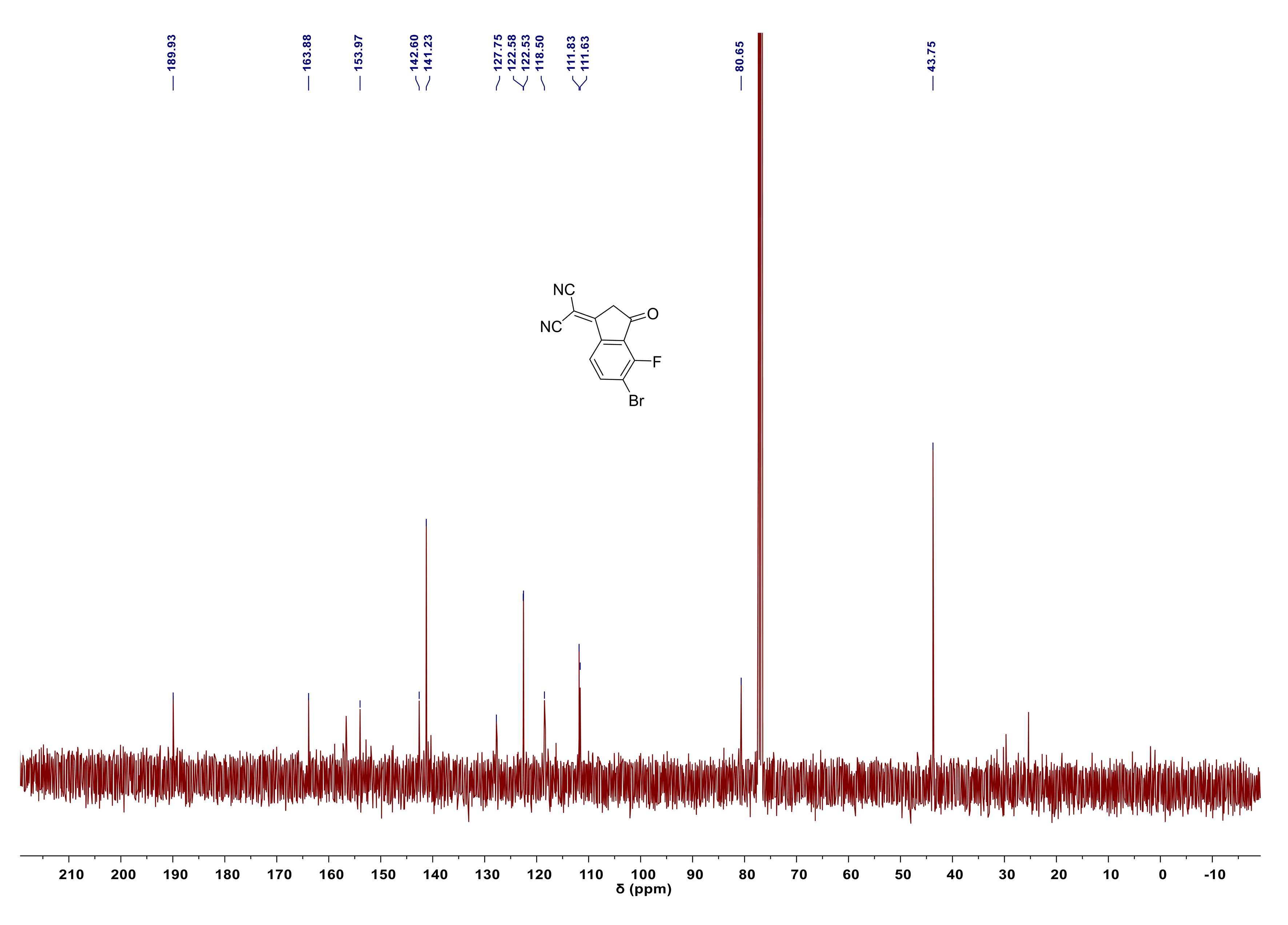
**

**Figure S8.** ^13^C NMR spectrum of **IC-FBr** (101 MHz, CDCl_3_): *δ* 189.93, 163.88, 153.97, 142.60, 141.23, 122.58, 122.53, 118.50, 111.83, 111.63, 43.75.


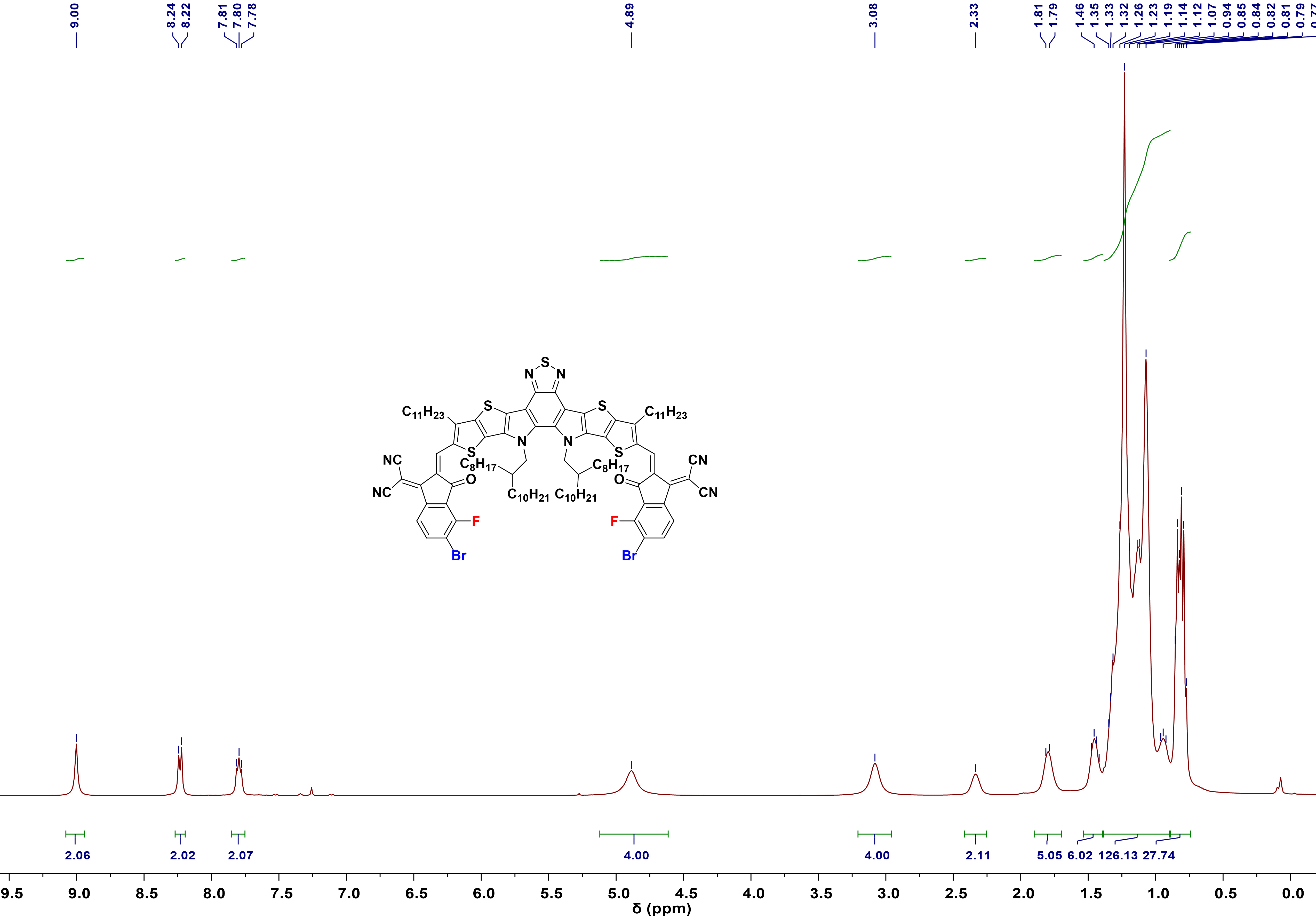


**Figure S9**. ^1^H NMR spectrum of **Y-OD-FBr** (400 MHz, CDCl_3_): δ 9.00 (s, 2H), 8.23 (d, J = 8.3 Hz, 2H), 7.80 (t, J = 7.1 Hz, 2H), 4.89 (s, 4H), 3.08 (s, 4H), 2.33 (s, 2H), 1.90 – 1.70 (m, 4H), 1.46 (t, J = 7.6 Hz, 4H), 1.39 – 0.89 (m, 92H), 0.81 (dq, J = 14.8, 7.5, 7.0 Hz, 18H).


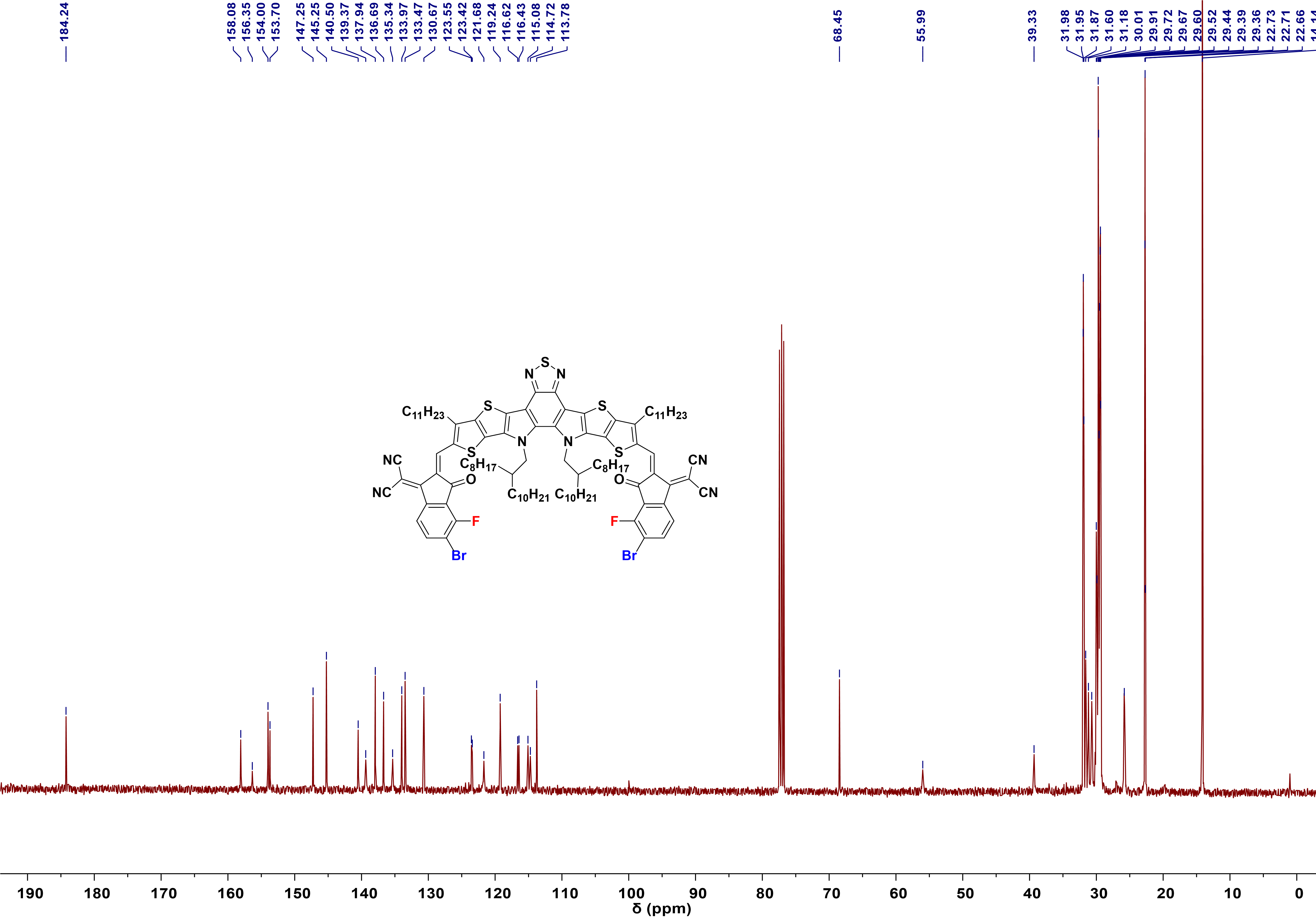


**Figure S10**. ^13^C NMR spectrum of **Y-OD-FBr** (101 MHz, CDCl_3_): δ 184.24, 158.08, 154.00, 153.70, 147.25, 145.25, 140.50, 139.37, 137.94, 136.69, 135.34, 133.97, 133.47, 130.67, 123.55, 123.42, 121.68, 119.24, 116.62, 116.43, 115.08, 114.72, 113.78, 68.45, 55.99, 39.33, 31.98, 31.95, 31.87, 31.60, 31.18, 30.69, 30.01, 29.91, 29.72, 29.67, 29.60, 29.52, 29.44, 29.39, 29.36, 25.82, 22.73, 22.71, 22.66, 14.14.


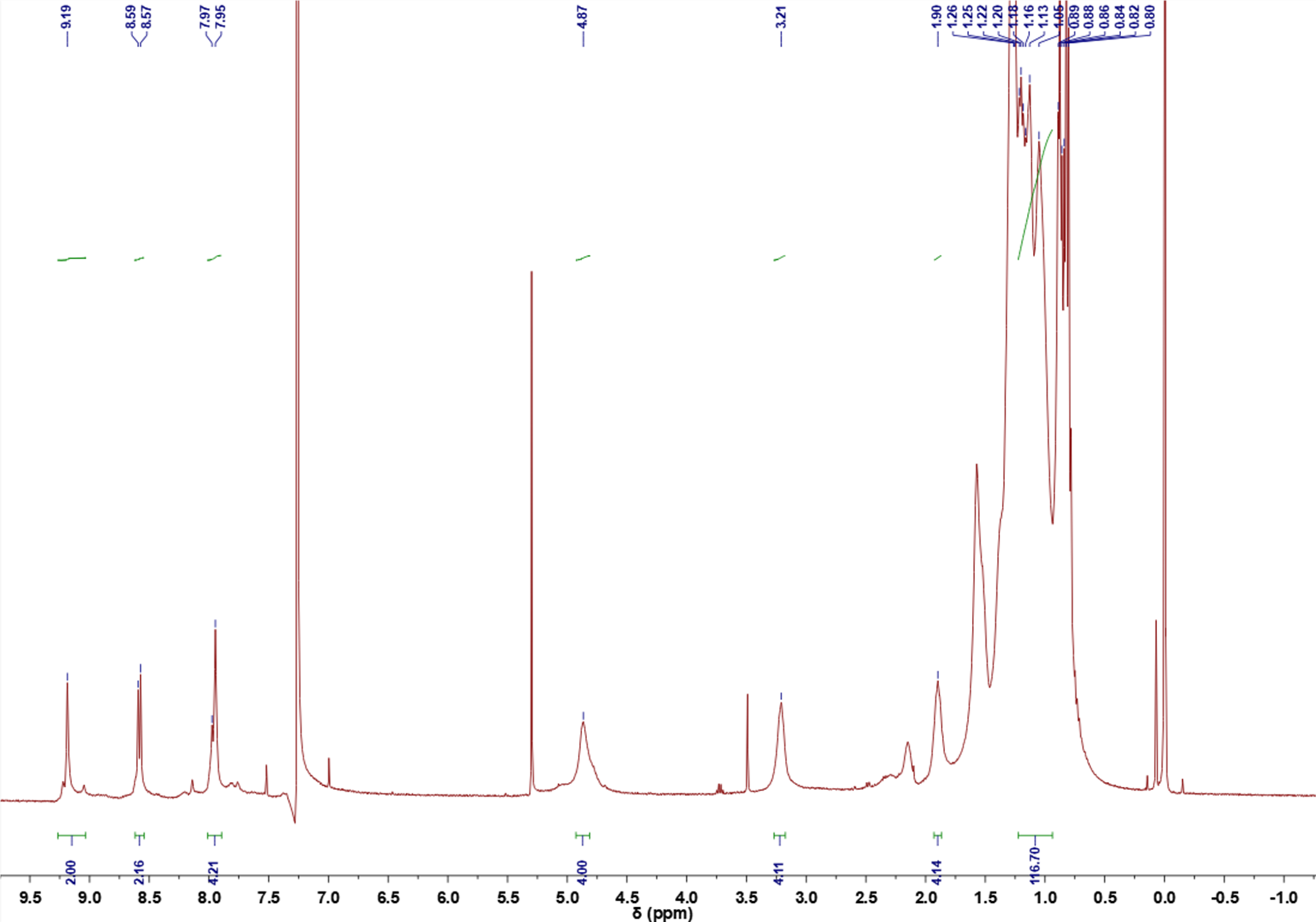


**Figure S11**. ^1^H NMR spectrum of **SP2** (400 MHz, CDCl_3_).

**
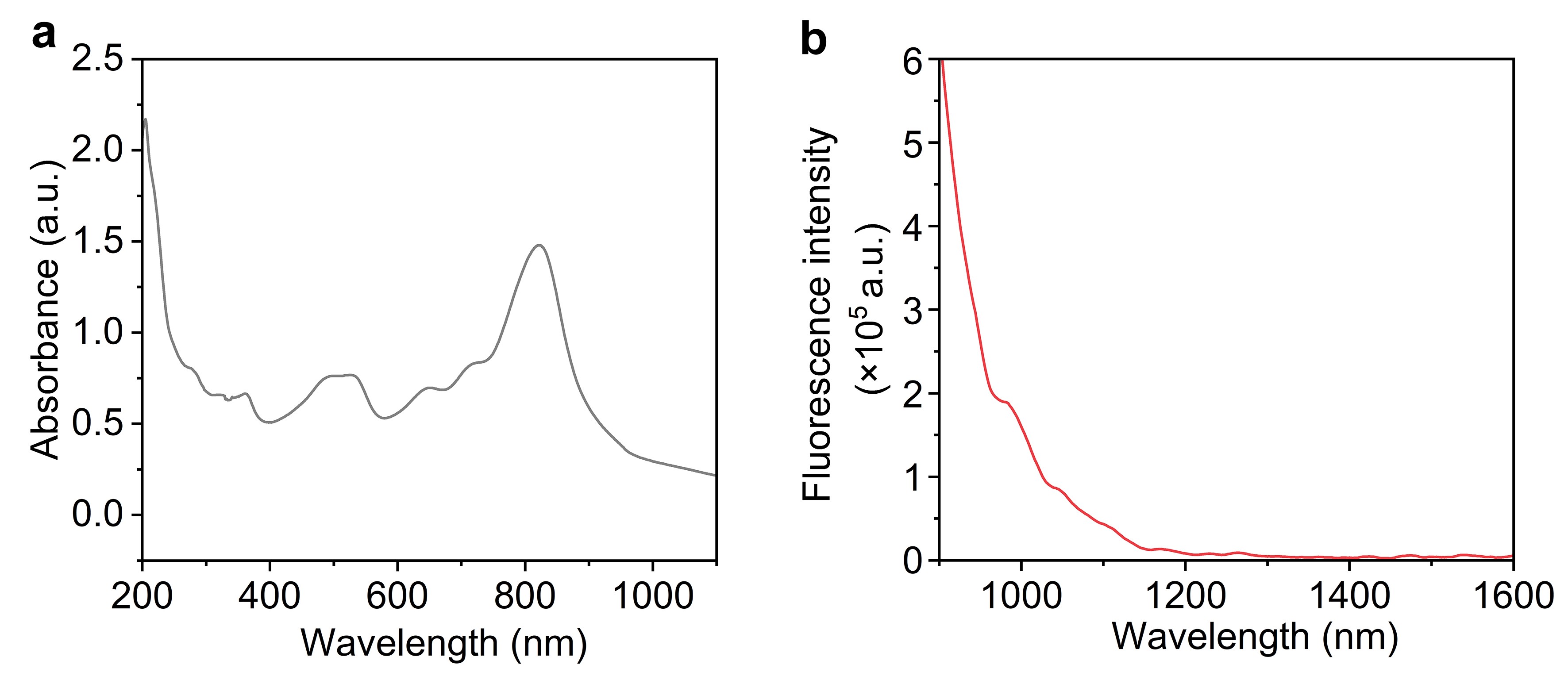
**

**Figure S12.** (a) UV-vis-NIR spectrum and (b) fluorescence spectrum (λ_ex_ = 808 nm) of SP2.


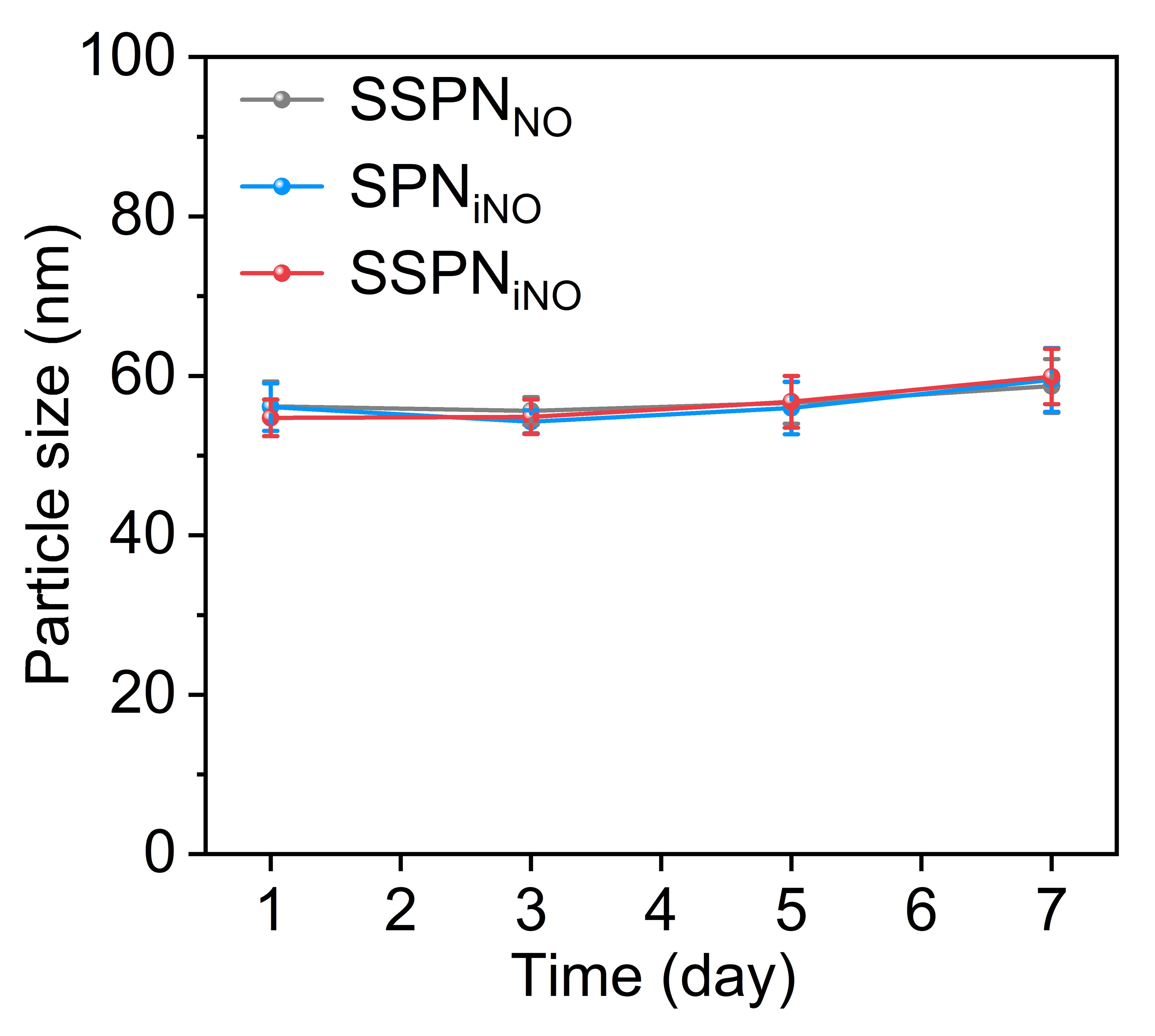


**Figure S13.** Particle sizes of SSPN_iNO_, SPN_iNO_ and SSPN_NO_ after different incubation times (n = 5).


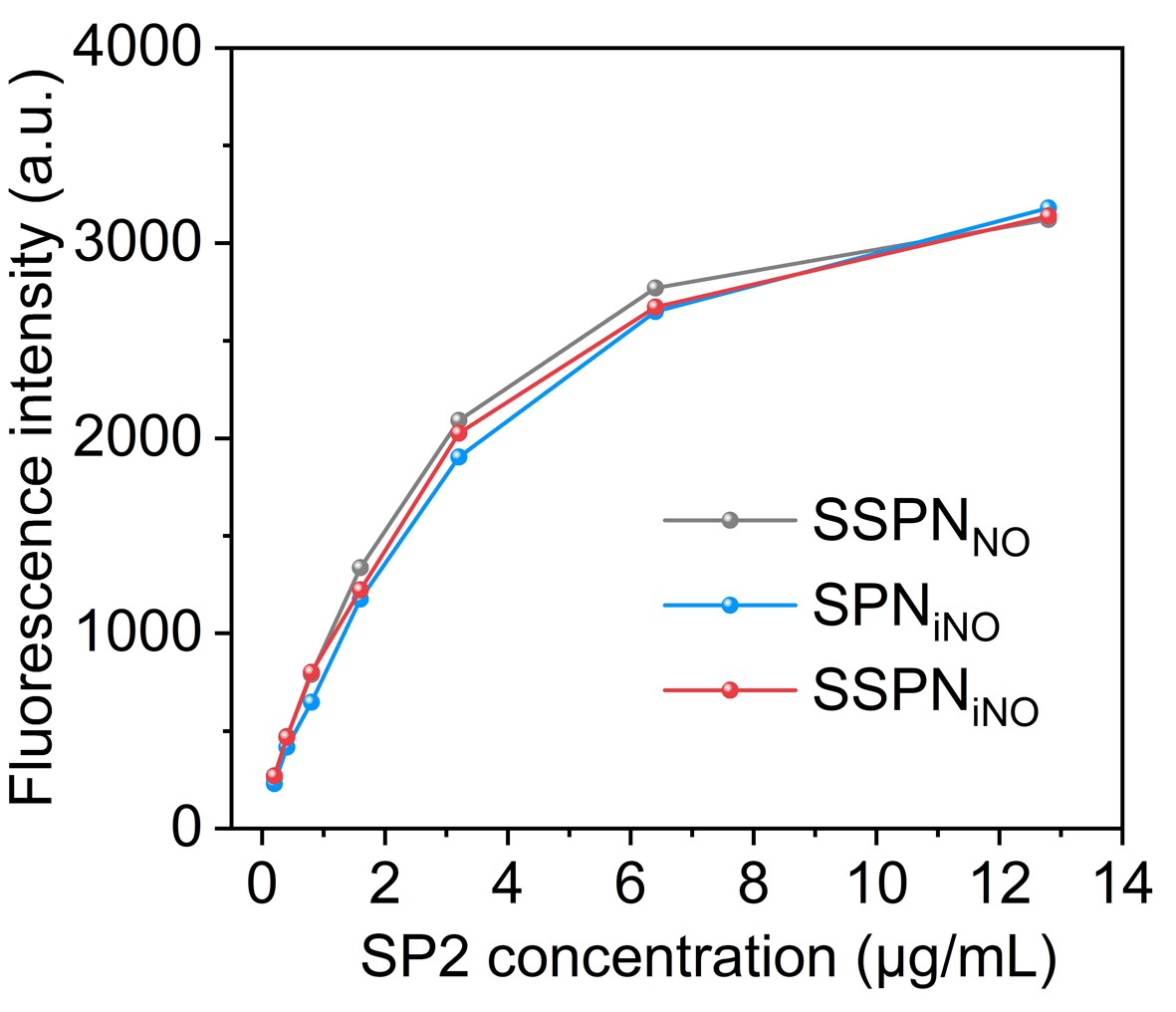


**Figure S14.** NIR-II fluorescence intensities of SSPN_iNO_, SPN_iNO_ and SSPN_NO_ at different concentrations of SP2. Excitation length: 808 nm.


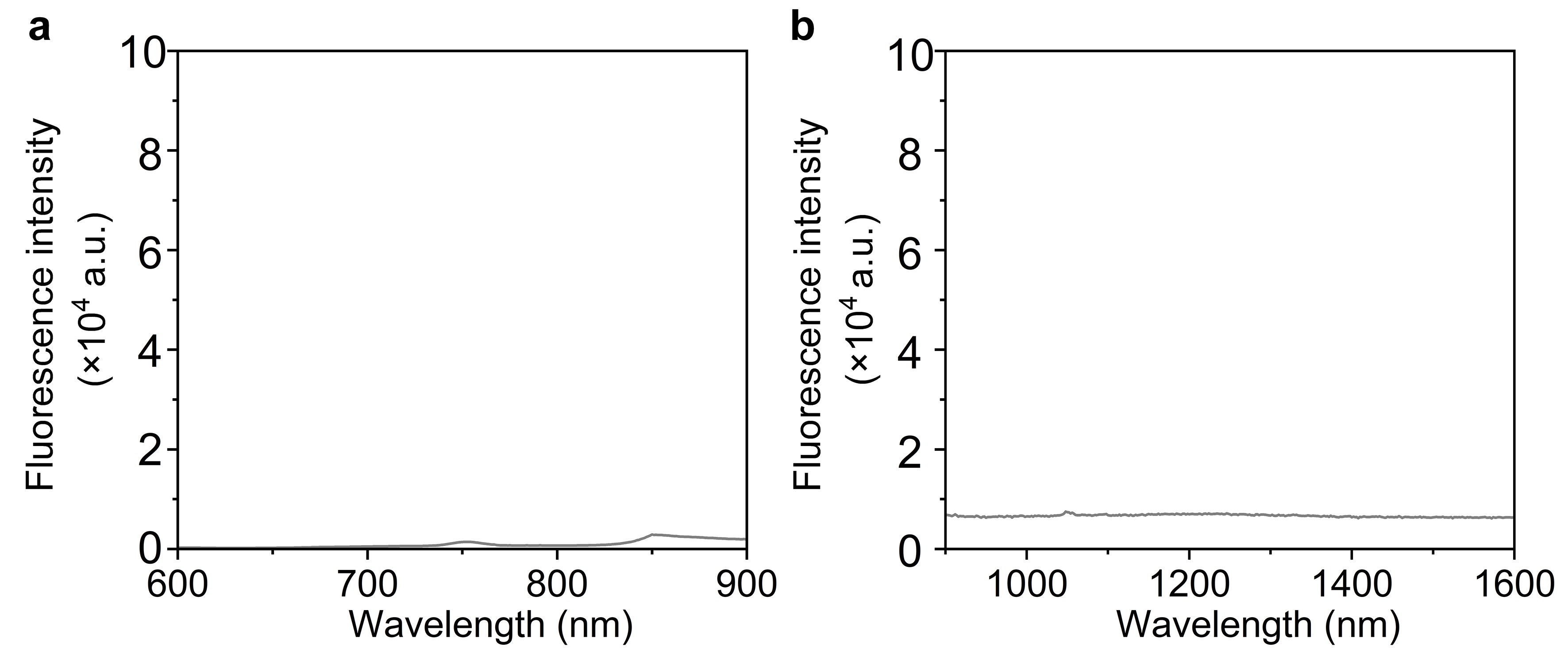


**Figure S15.** Fluorescence spectra of SP1 in (a) NIR-I window (λ_ex_ = 500 nm) and (b) NIR-II window (λ_ex_ = 808 nm).


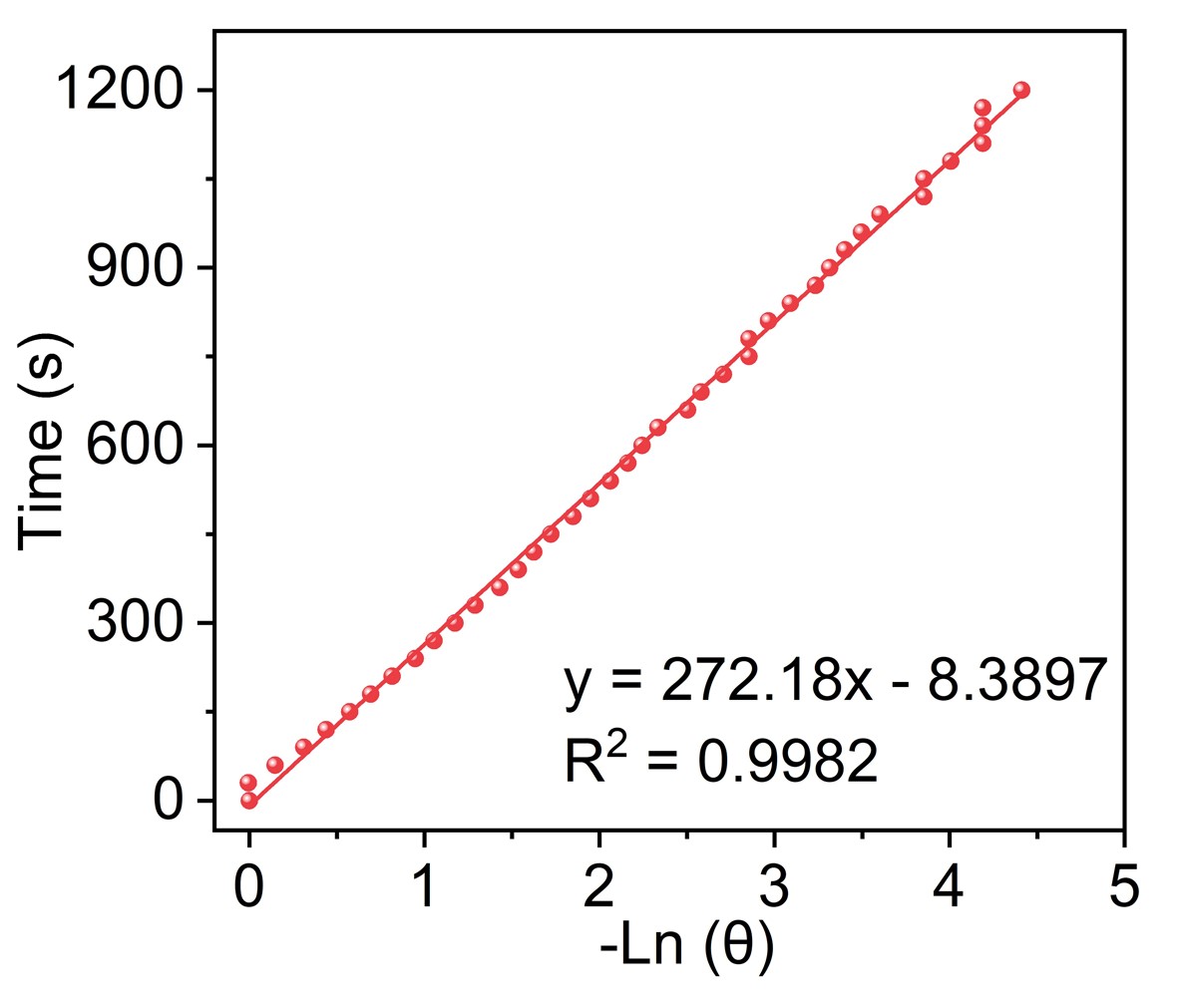


**Figure S16.** Linear time versus -Ln(θ) from the cooling period in Figure 3e.


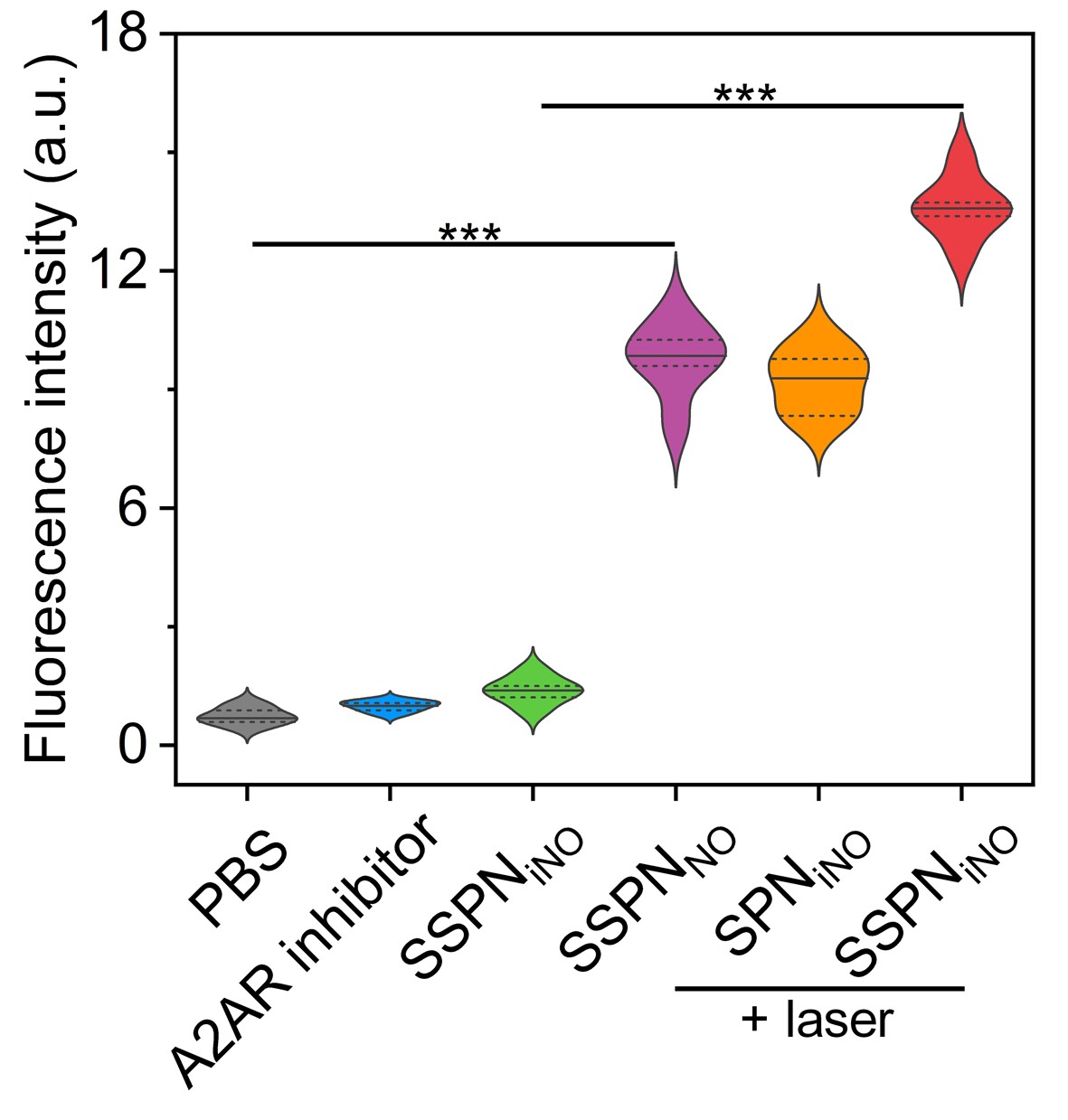


**Figure S17.** Analysis of NO generation in C6 cells (n = 6). All data are presented as median ± IQR (^***^*p* < 0.001, ANOVA with Turkey’s post-hoc tests).


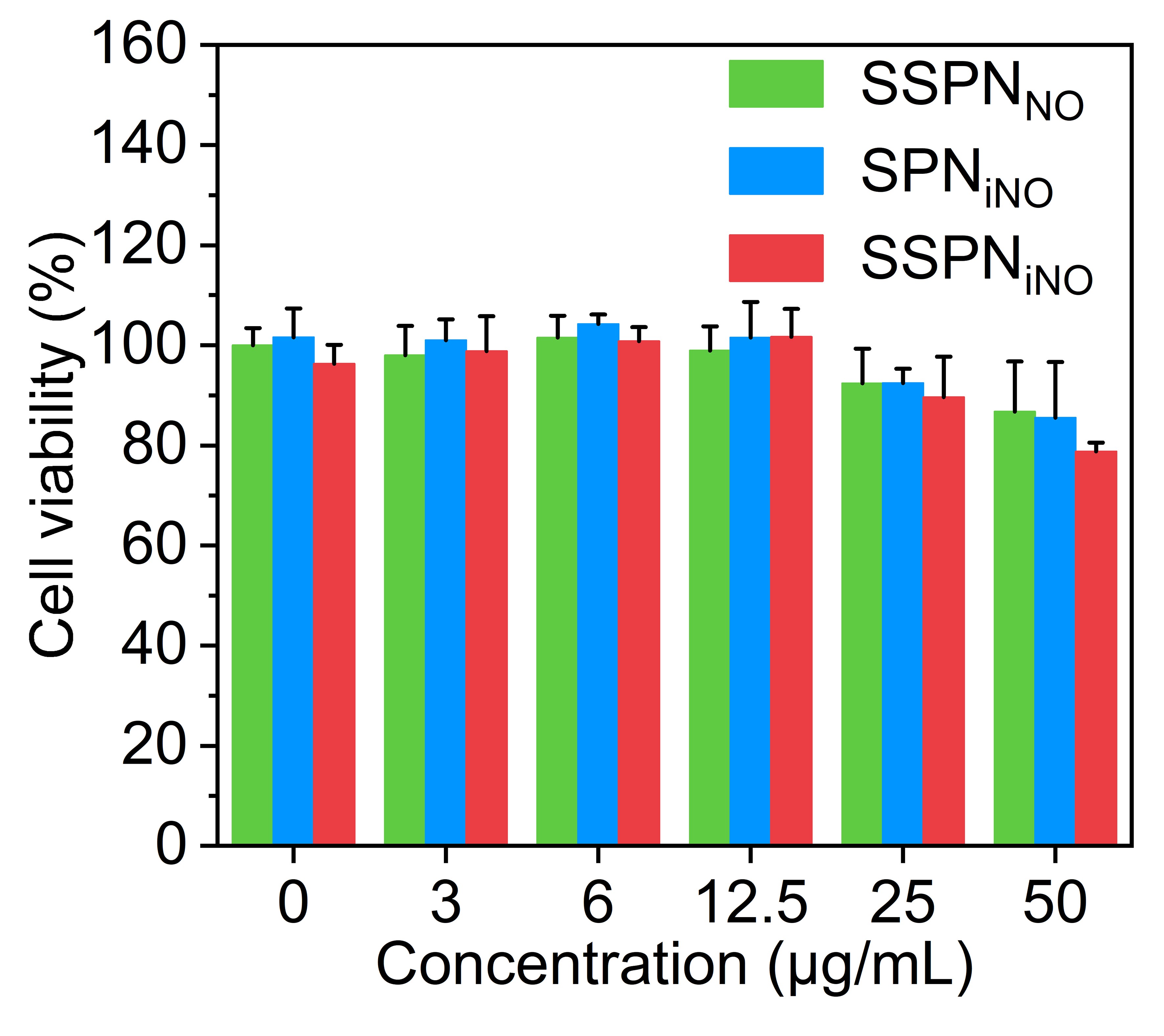


**Figure S18.** Cell viability of C6 cells after treatments with SSPN_iNO_, SPN_iNO_ and SSPN_NO_ at various concentrations (n = 3). All data are presented as mean ± SD.


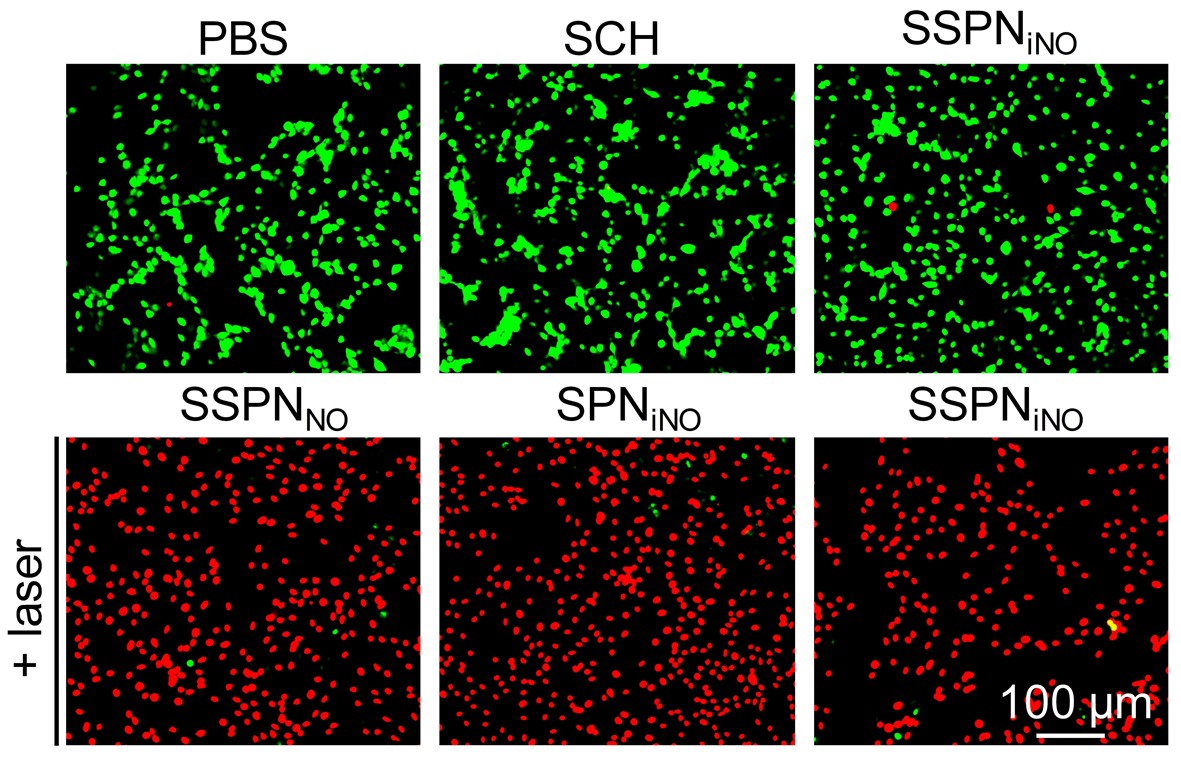


**Figure S19.** Fluorescence images of calcein-AM/PI stained C6 cells after treatments with SSPN_iNO_, SPN_iNO_ and SSPN_NO_ (50 μg/mL) with or without laser irradiation (1064 nm, 1.0 W/cm^2^, 5 min).


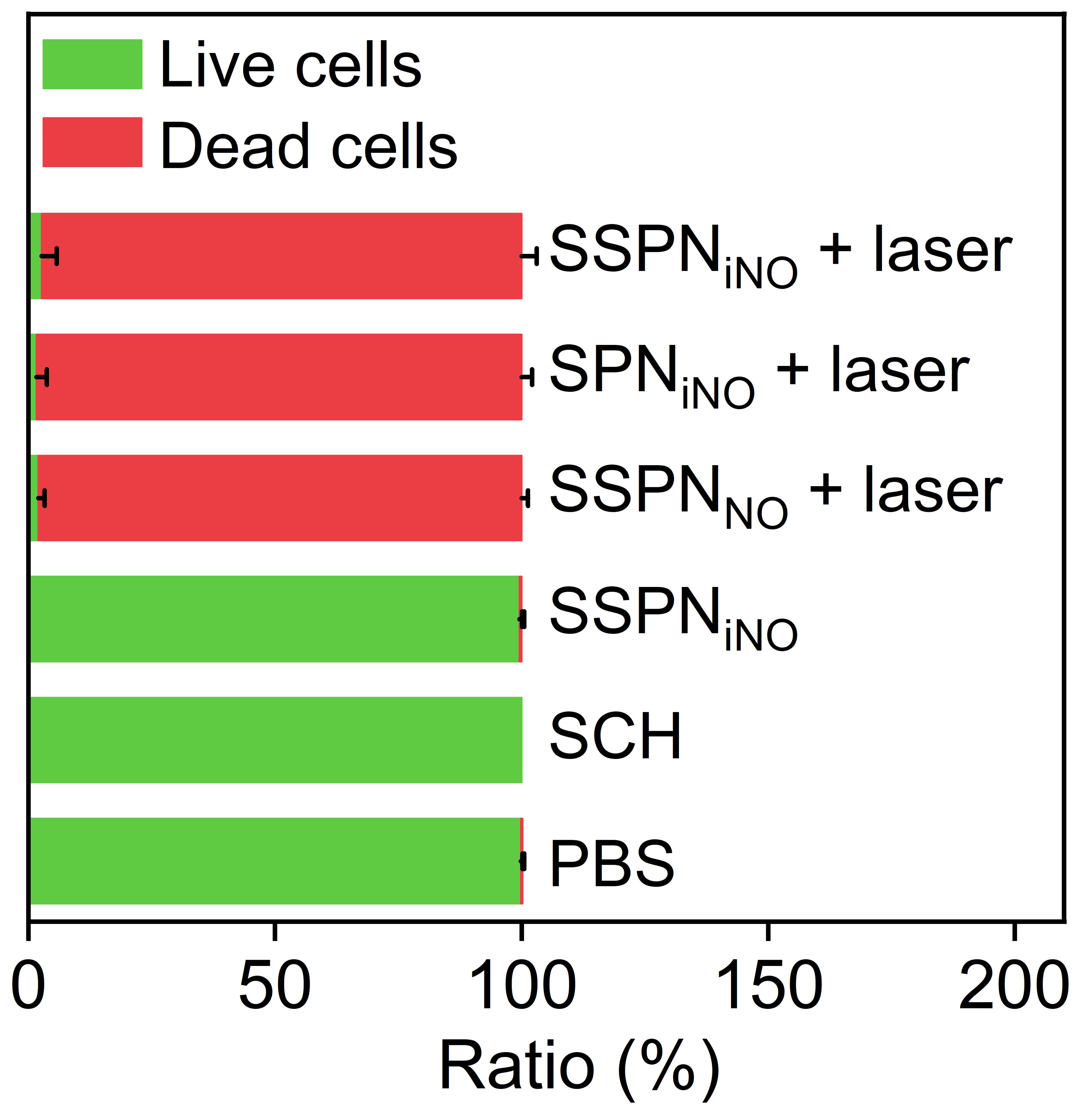


**Figure S20.** Analysis of live and dead cell ratios after various treatments (n = 4). All data are presented as mean ± SD.


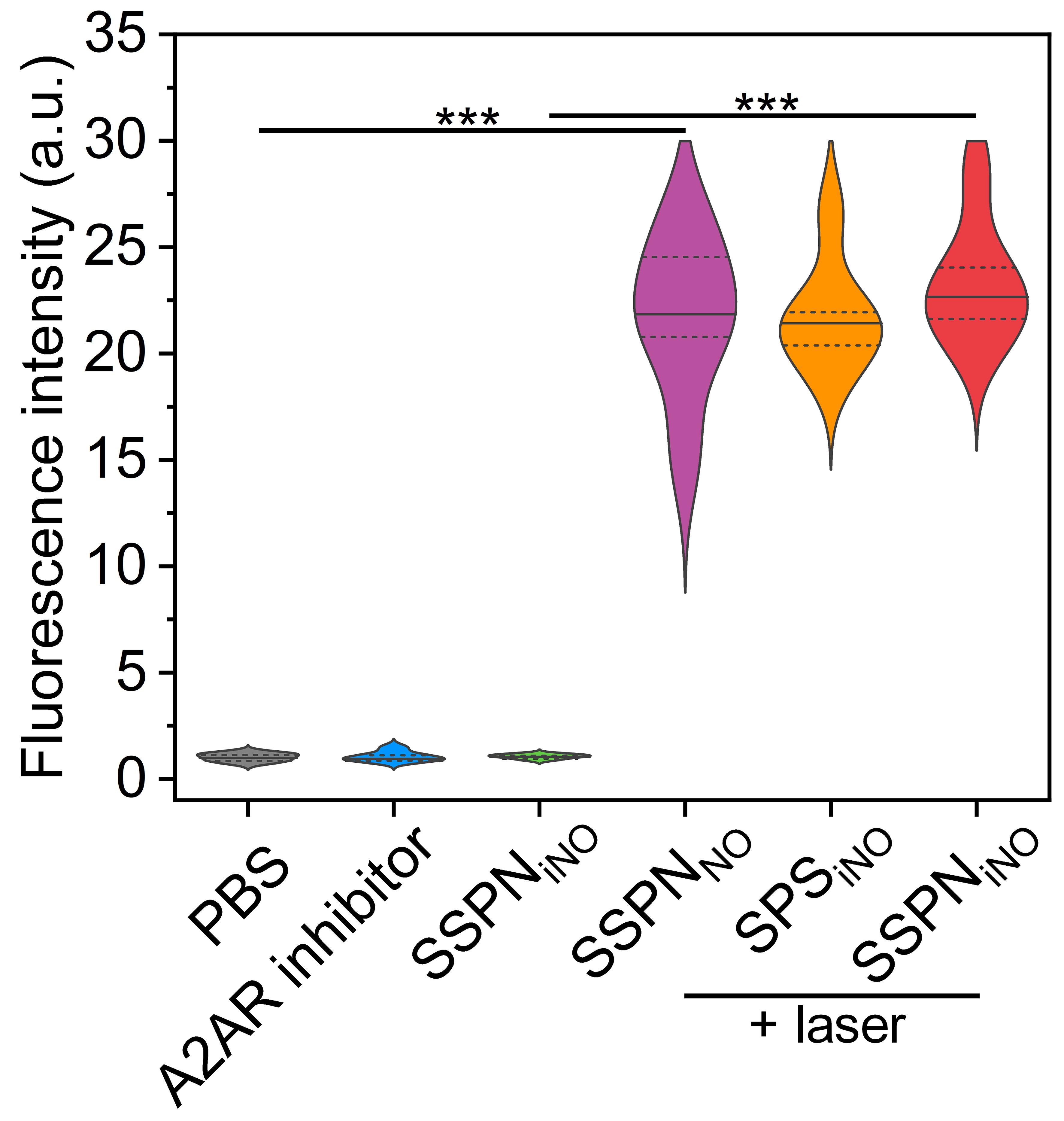


**Figure S21.** Analysis of CRT expression levels in C6 cells after treatments with SSPN_iNO_, SPN_iNO_ and SSPN_NO_ (50 μg/mL) with or without laser irradiation (n = 6). All data are presented as median ± IQR (^***^*p* < 0.001, ANOVA with Turkey’s post-hoc tests).


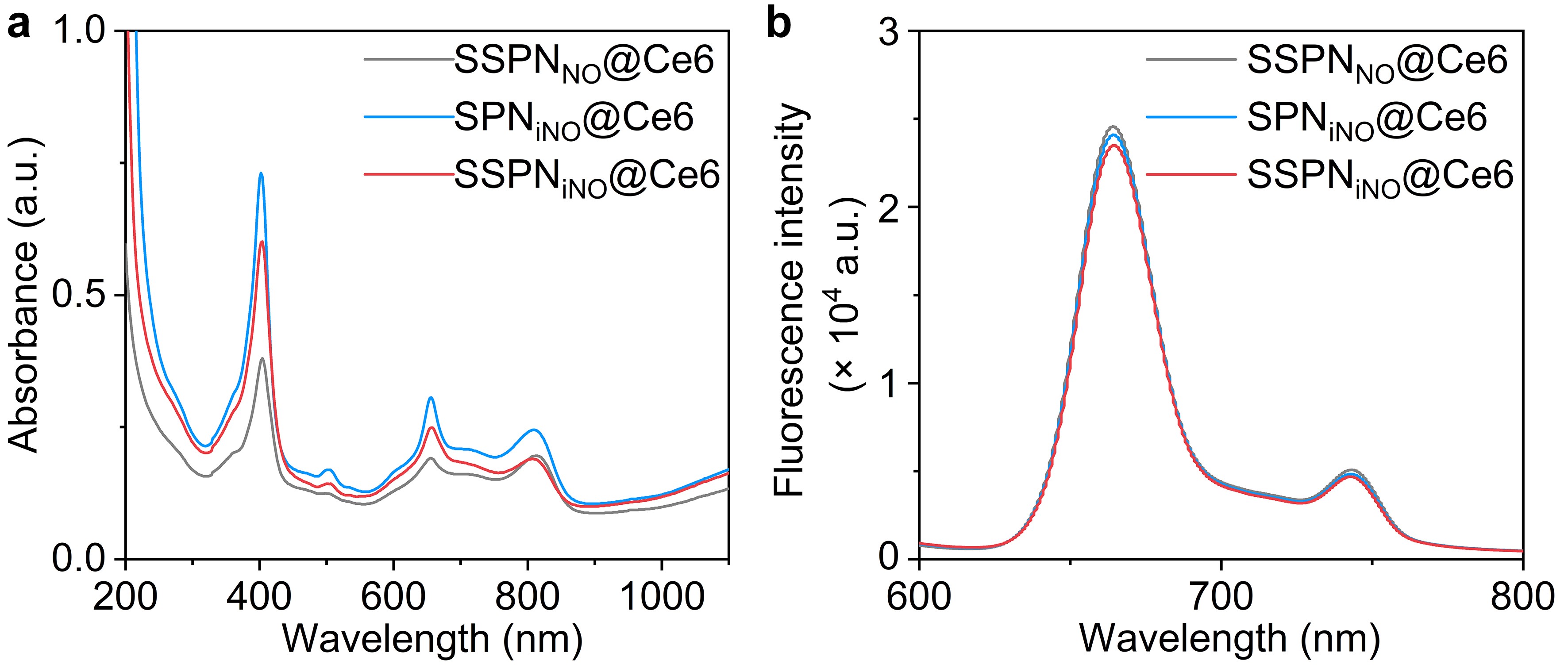


**Figure S22.** (a) UV-vis spectra and (b) fluorescence spectra (λ_ex_ = 495 nm) of SSPN_iNO_, SPN_iNO_ and SSPN_NO_ with Ce6 labeling.

**
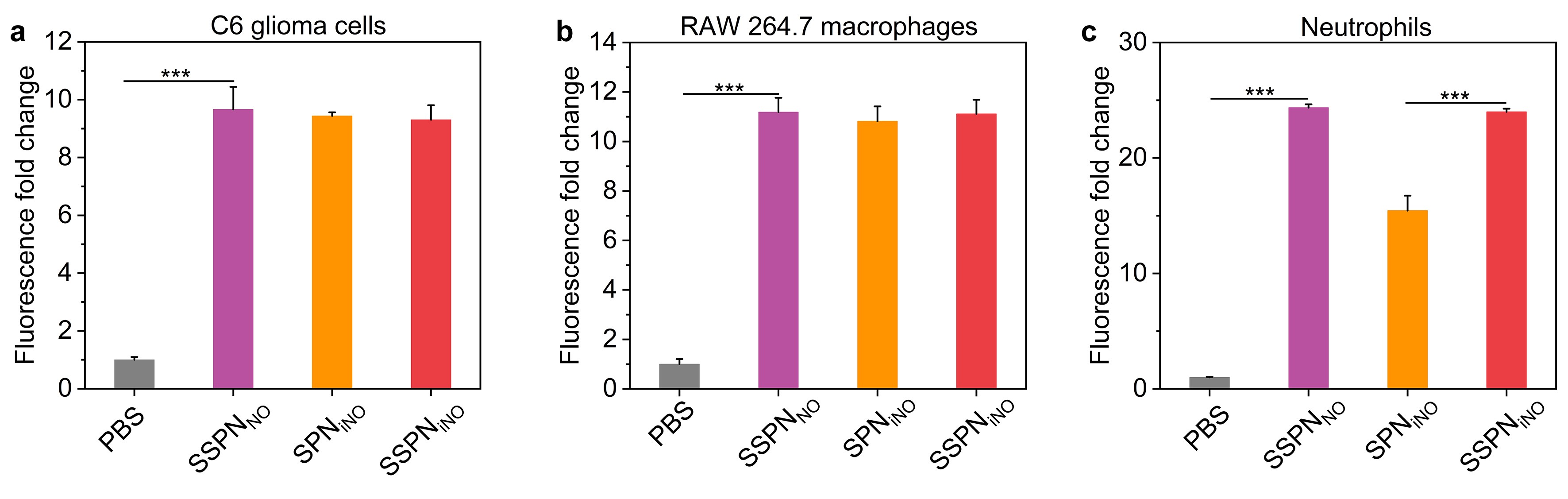
**

**Figure S23.** Uptake analysis of Ce6-labeled SSPN_iNO_, SPN_iNO_ and SSPN_NO_ by (a) C6 glioma cells, (b) RAW 264.7 macrophages and (c) neutrophils (n = 3). All data are presented as mean ± SD (^***^*p* < 0.001, ANOVA with Turkey’s post-hoc tests).


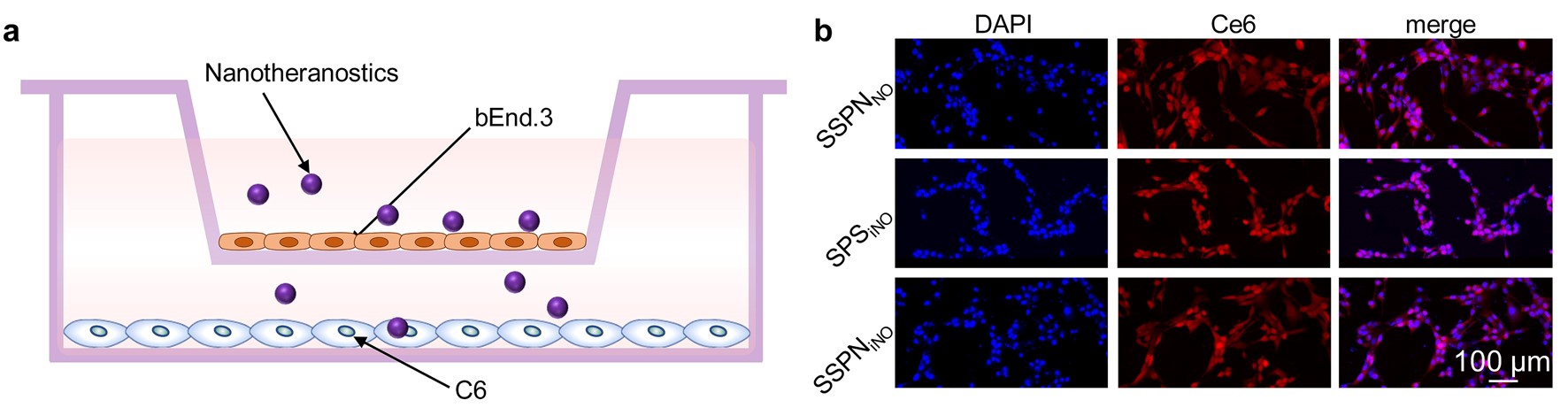


**Figure S24.** (a) Schematic illustration of the penetration of nanotheranostics across *in vitro* BBB model on transwell systems. (b) Confocal fluorescence images of C6 cells in transwell systems after treatments with Ce6-labeled SSPN_NO_, SPN_iNO_ and SSPN_iNO_.

**
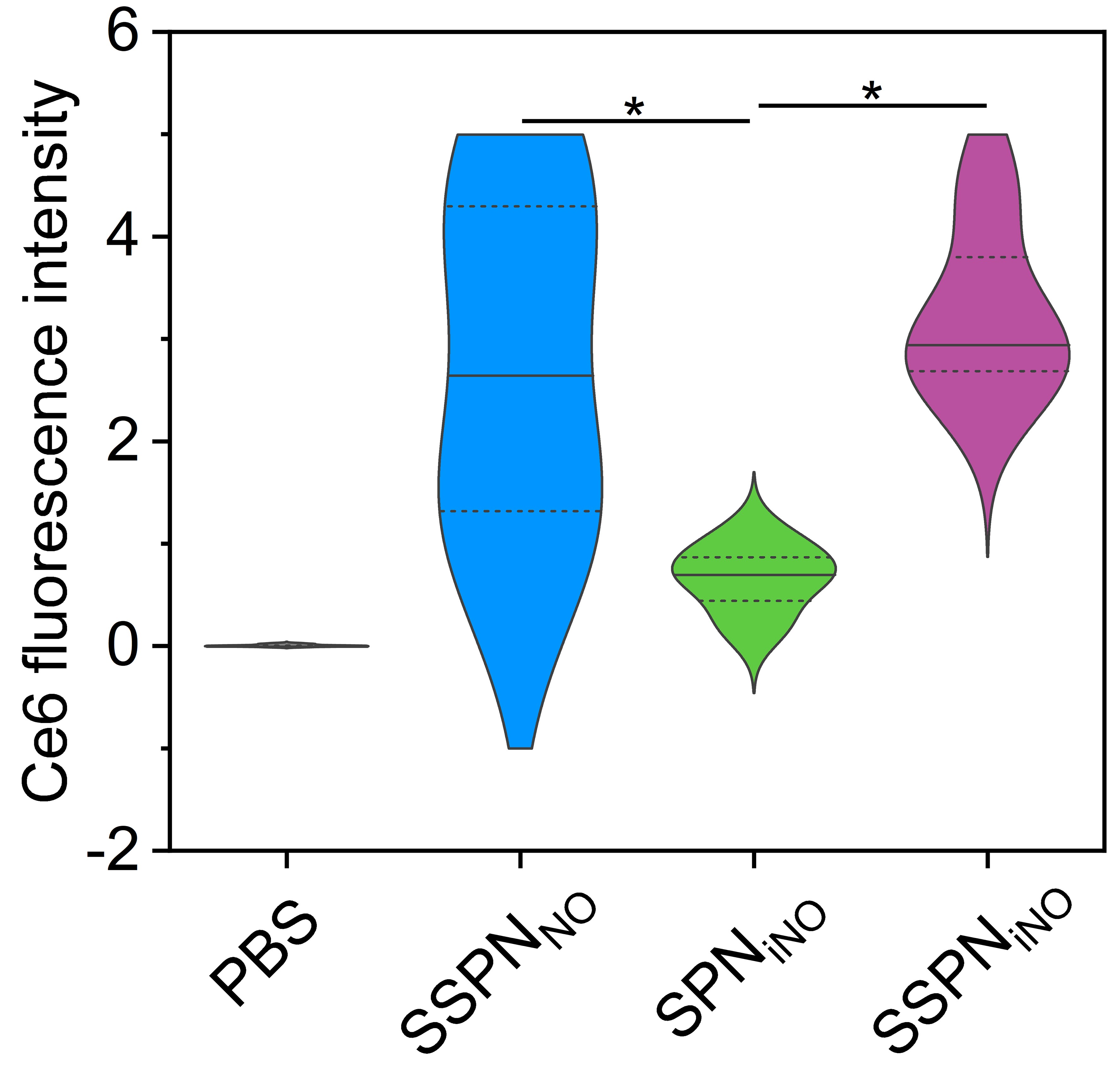
**

**Figure S25.** Analysis of fluorescence signal intensity of Ce6-labeled SSPN_iNO_, SPN_iNO_ and SSPN_NO_ in orthotopic tumor tissues (n = 4). All data are presented as median ± IQR (^*^*p* < 0.05, ANOVA with Turkey’s post-hoc tests).


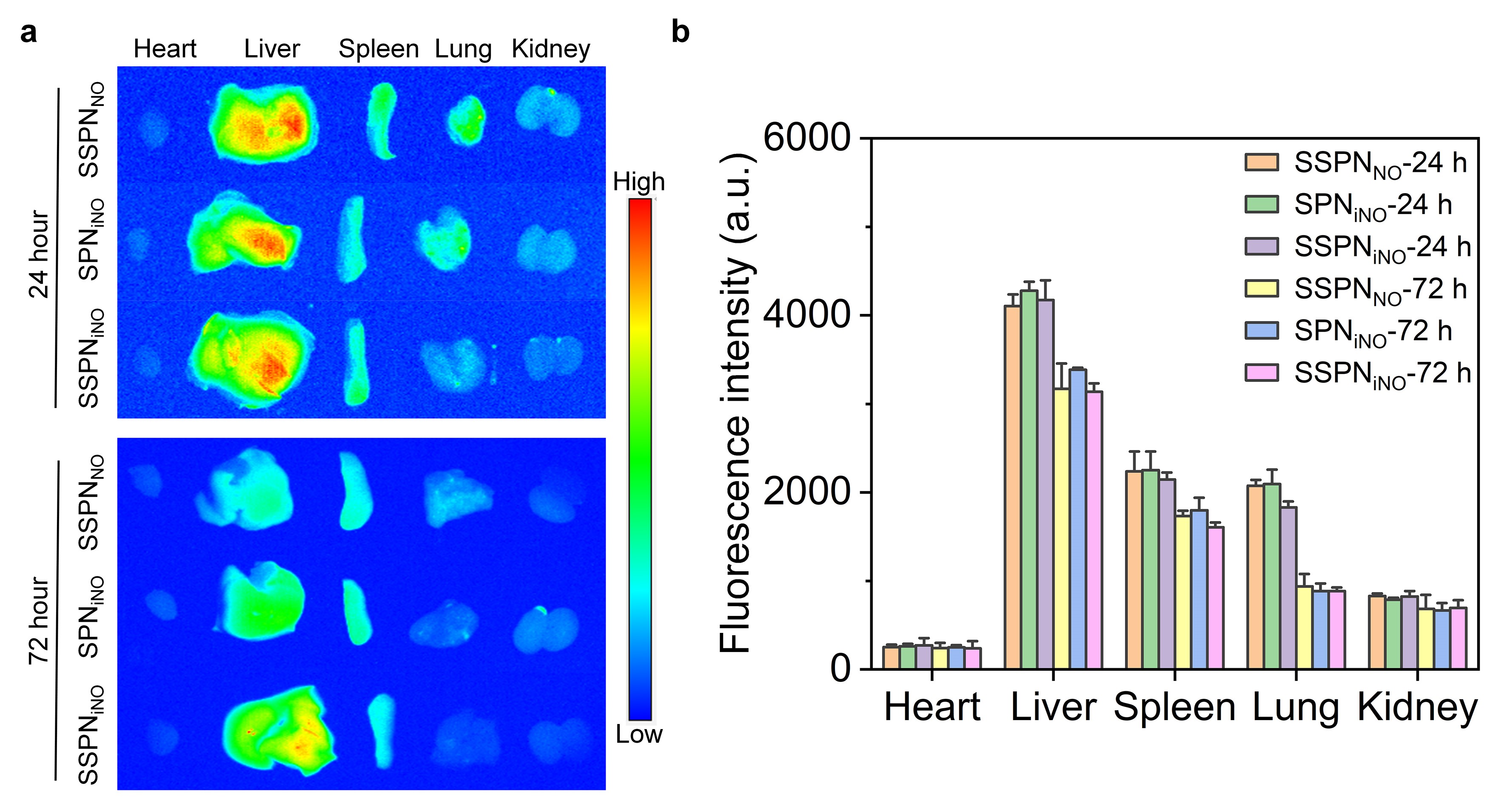


**Figure S26.** (a) Biodistribution analysis of nanotheranostics in living mice after different times. (b) Quantitative analysis of fluorescence intensity in heart, liver, spleen, lung and kidney (n = 5). All data are presented as mean ± SD.


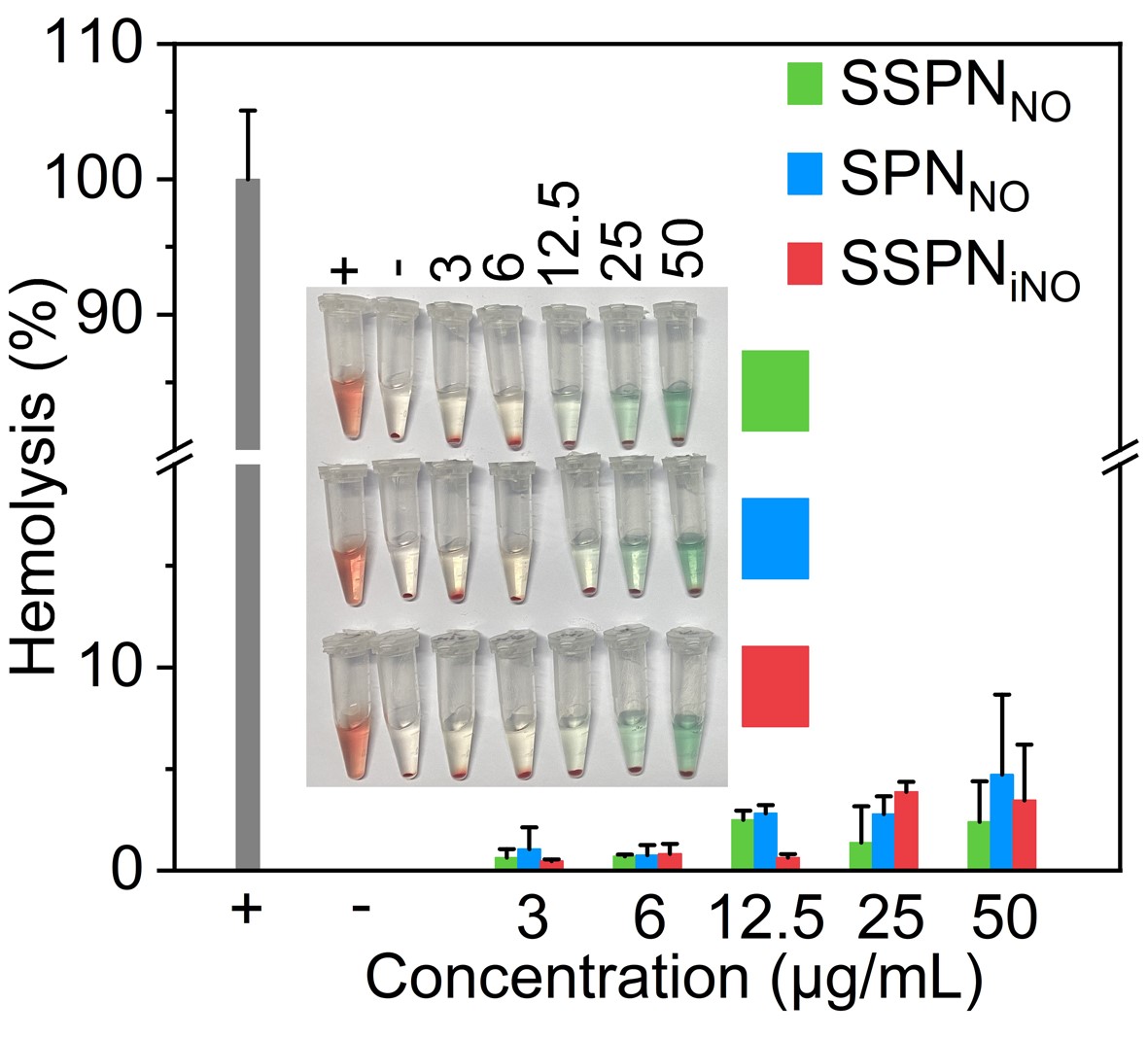


**Figure S27.** Hemolytic assay of SSPN_NO_, SPN_iNO_ and SSPN_iNO_ using red blood cells (n = 3). All data are presented as mean ± SD.


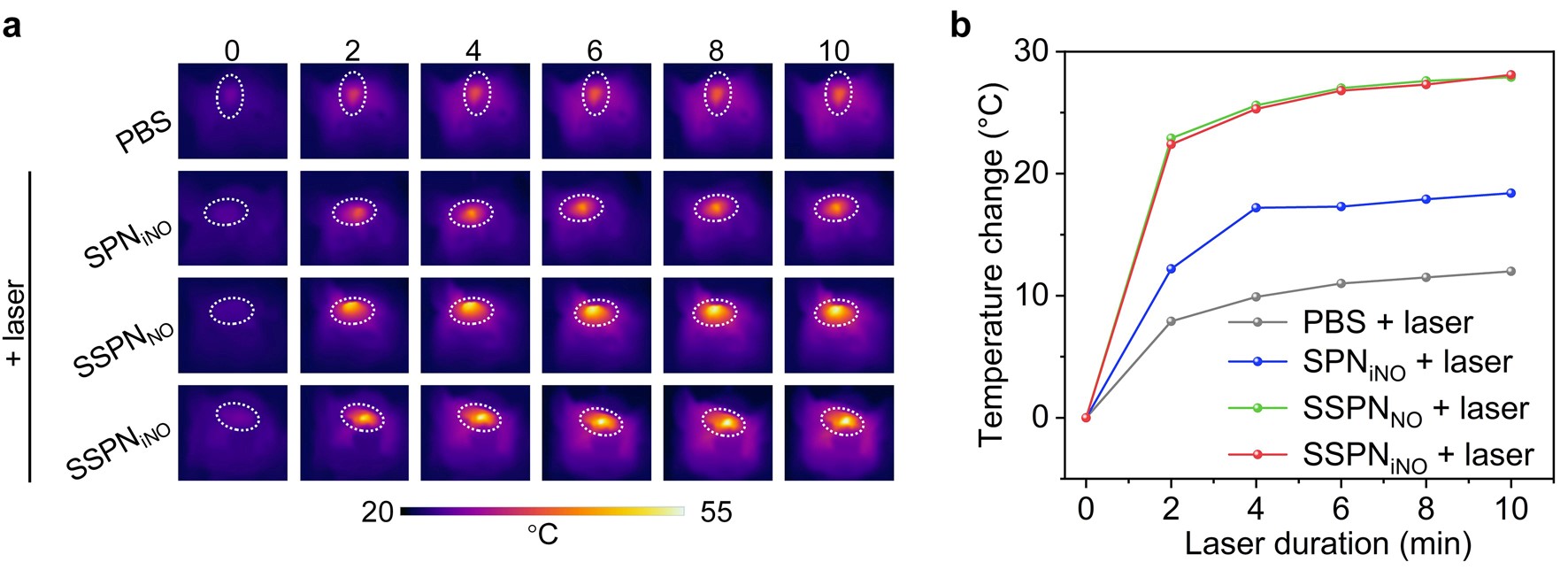


**Figure S28.** (a) In vivo photothermal images of orthotopic GBM-bearing mice under 1064 nm laser irradiation (1.0 W/cm^2^, 10 min). (b) Temperature changes of tumor sites as a function of laser time in different groups.


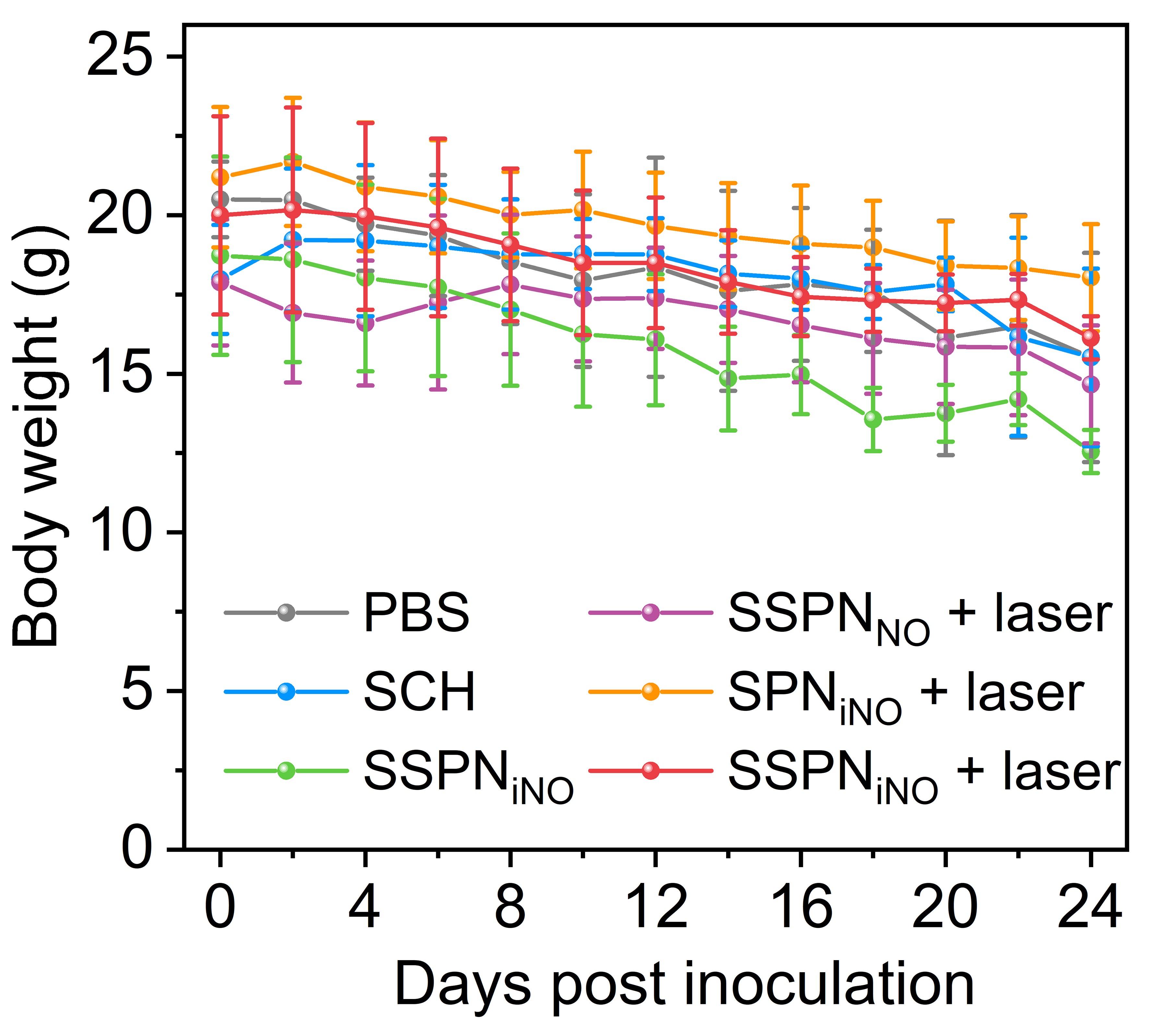


**Figure S29.** Body weights of glioma-bearing mice after different treatments (n = 6). All data are presented as mean ± SD.


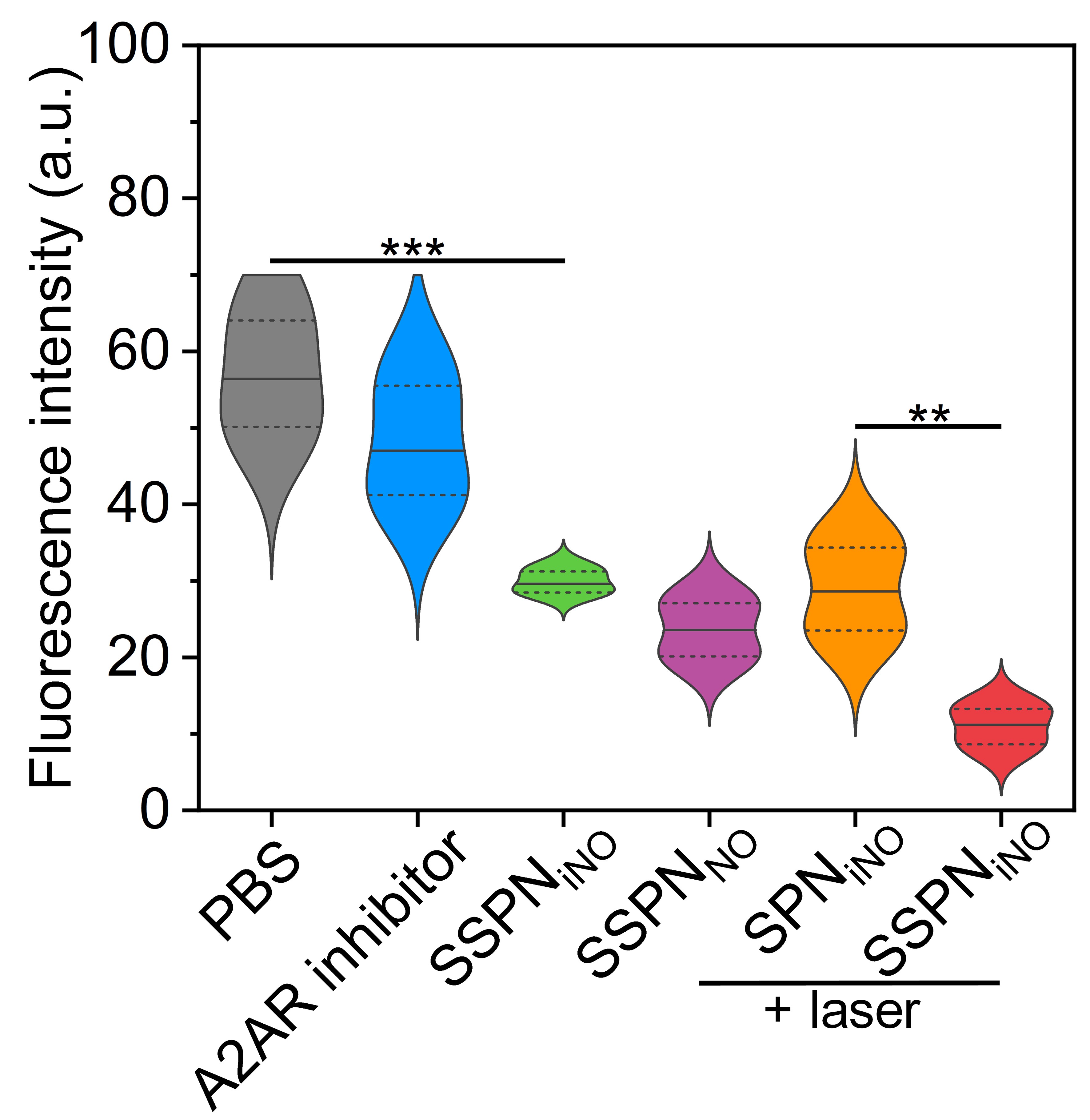


**Figure S30.** Analysis of Ki-67 staining signal intensity of tumors (n = 4). All data are presented as median ± IQR (^**^*p* < 0.01, ^***^*p* < 0.001, ANOVA with Turkey’s post-hoc tests).


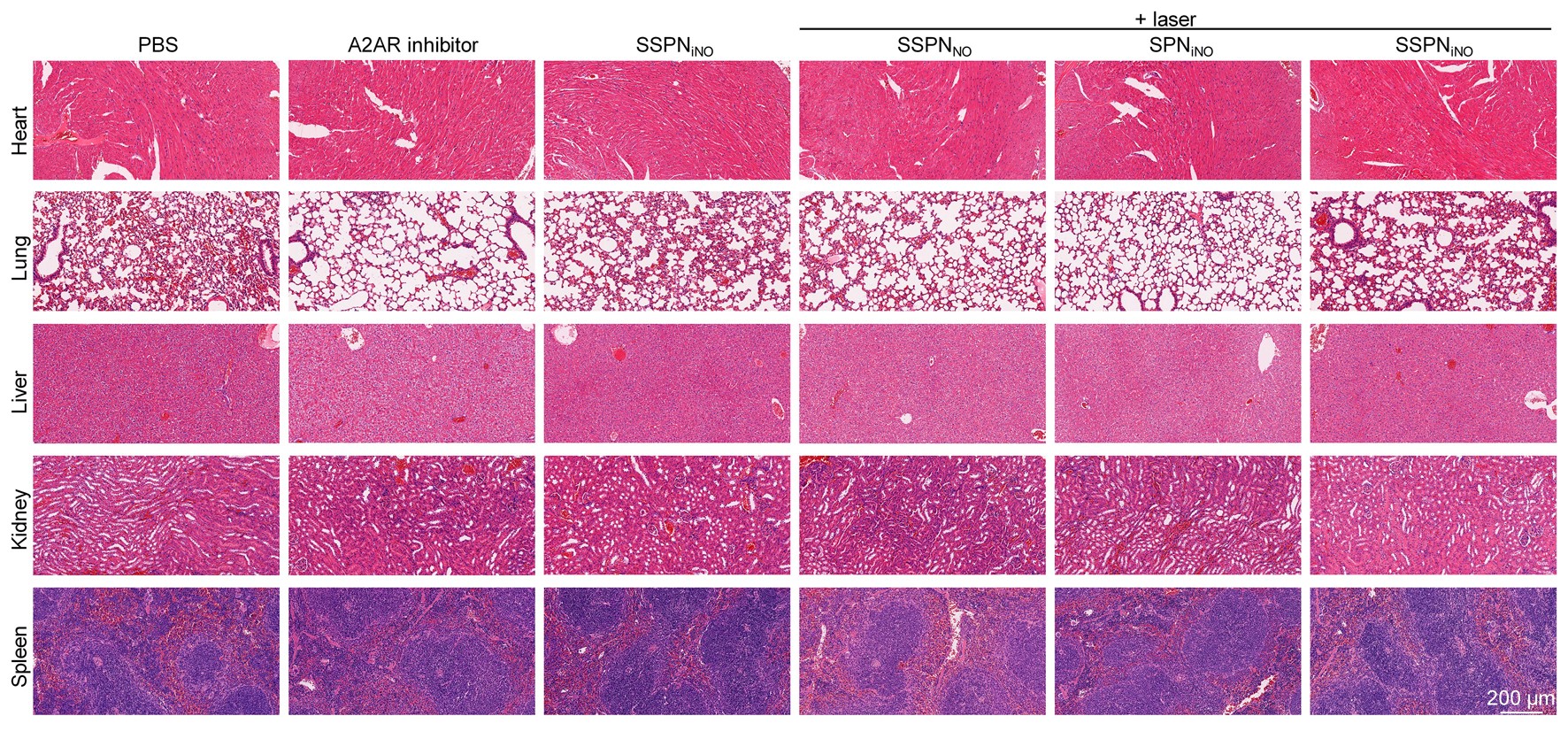


**Figure S31.** H&E staining images of heart, lung, liver, kidney and spleen excised from the mice in each group.


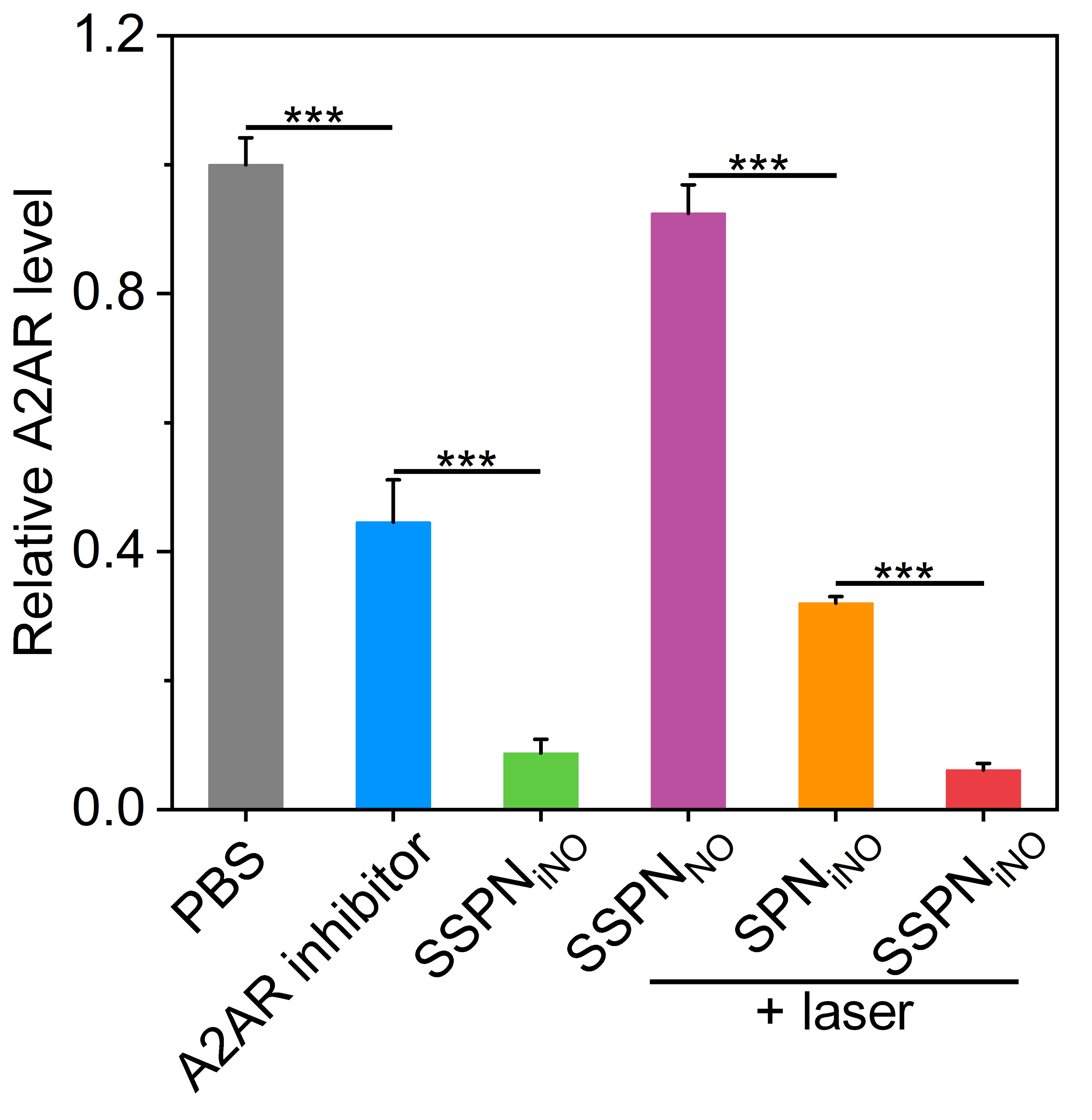


**Figure S32.** Analysis of A2AR expression levels in orthotopic GBM tumors (n = 4). All data are presented as mean ± SD (^***^*p* < 0.001, ANOVA with Turkey’s post-hoc tests).


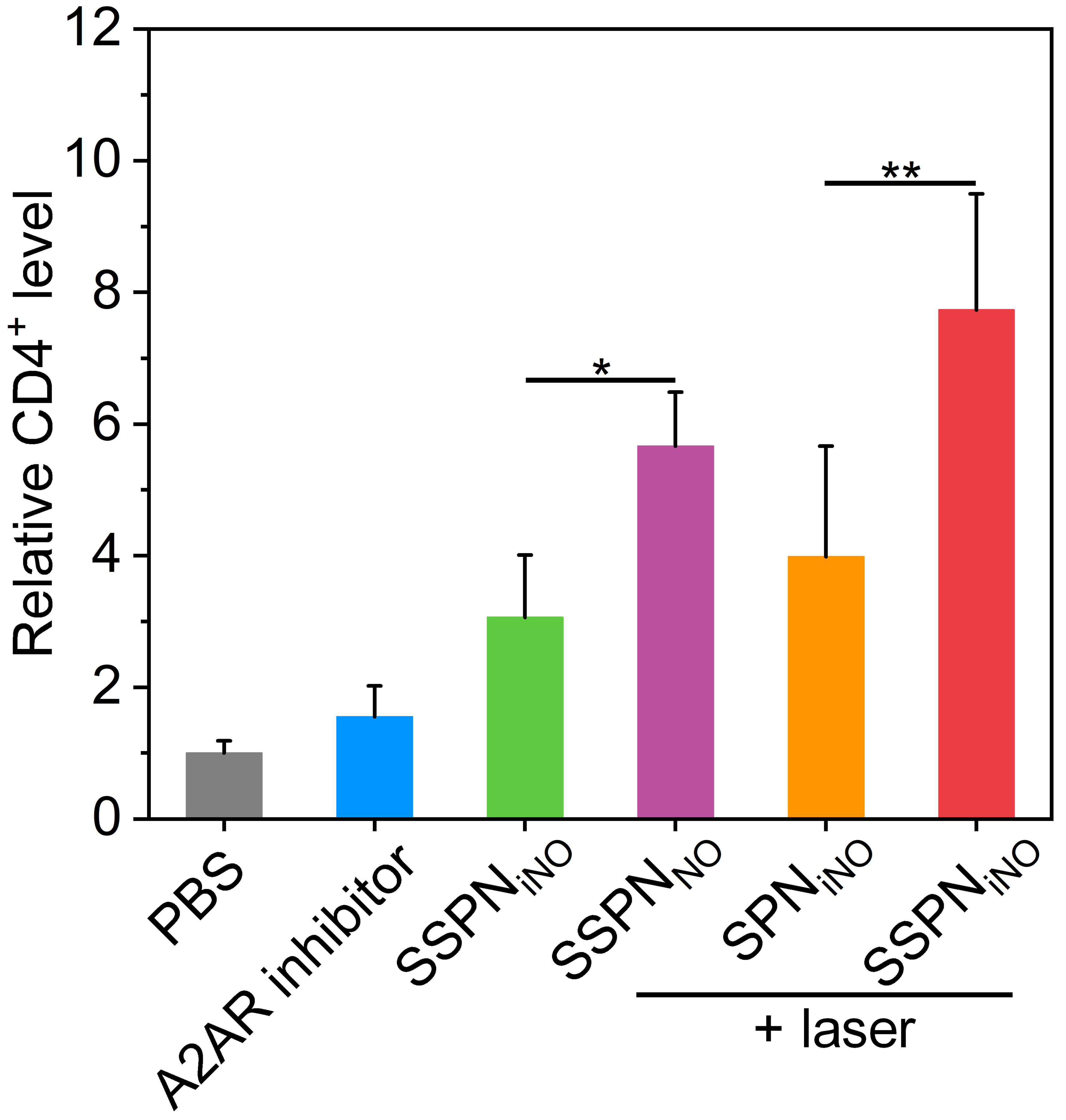


**Figure S33.** Analysis of CD4^+^ expression in orthotopic GBM tumors (n = 4). All data are presented as mean ± SD (^*^*p* < 0.05, ^**^*p* < 0.01, ANOVA with Turkey’s post-hoc tests).


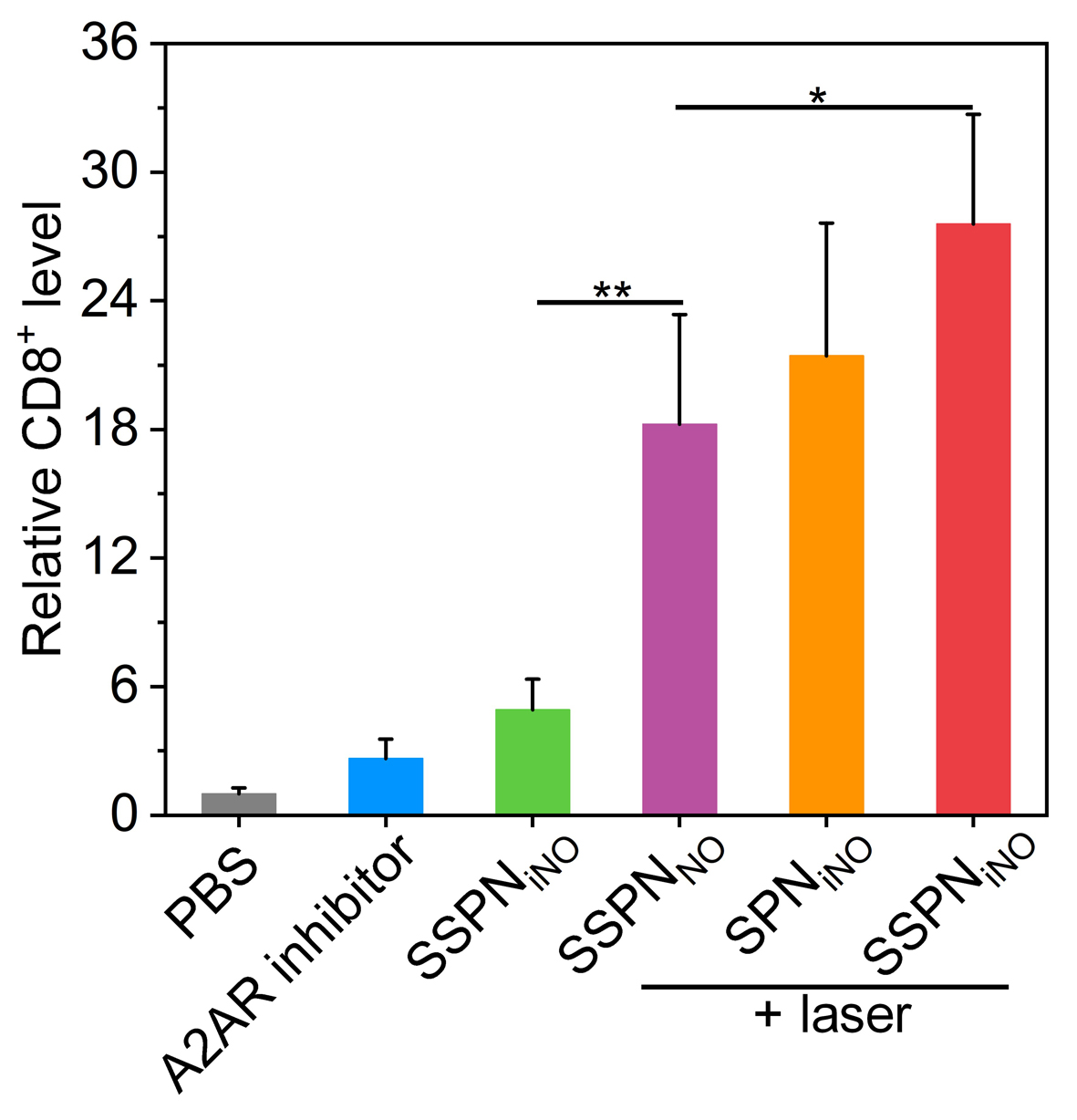


**Figure S34.** Analysis of CD8^+^ expression in orthotopic GBM tumors (n = 4). All data are presented as mean ± SD (^*^*p* < 0.05, ^**^*p* < 0.01, ANOVA with Turkey’s post-hoc tests).


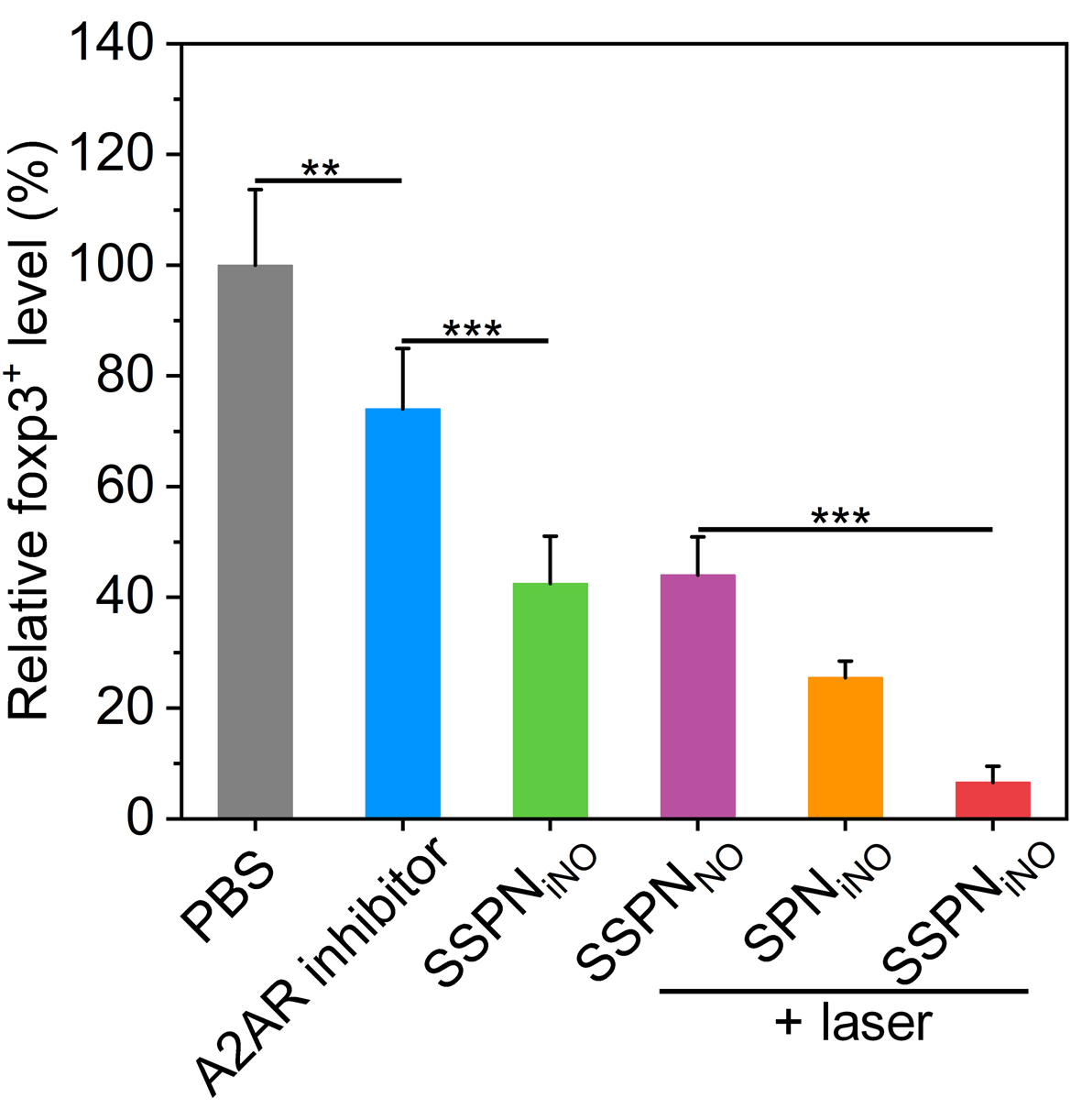


**Figure S35.** Analysis of Foxp3^+^ expression in orthotopic GBM tumors (n = 4). All data are presented as mean ± SD (^**^*p* < 0.01, ^***^*p* < 0.001, ANOVA with Turkey’s post-hoc tests).

# 2. Experimental Section

*Materials:* 1,2-Distearoyl-sn-glycero-3-phosphoethanolamine-poly(ethylene-glycol)-2000-N-hydroxysuccinimide (DSPE-PEG-NHS, Mw = 2 kDa) and 1,2-distearoyl-sn-glycero-3-phosphoethanolamine-N-[amino (polyethylene glycol)-2000] (DSPE-PEG-NH_2_, Mw = 2 kDa) were purchased from Ponsure Biological Co., Ltd (Shanghai, China). The A2AR inhibitor (SCH58261) was obtained from Selleck Chemicals (USA). Rabbit anti-A2AR antibody was purchased from Affinity Bioscience (Cincinnati, USA). Fluorescein isothiocyanate (FITC)-conjugated anti-Ly-6G antibody was obtained from Cell Signalling Technology (Danvers, MA). Other antibodies were purchased from Abcam Inc. (USA). Fetal bovine serum (FBS), Dulbecco’s modified Eagle medium (DMEM), and penicillin/streptomycin were purchased from Gibco (Grand Island, NY, USA). Sialic acid, calcein-AM/PI and cell counting kit-8 (CCK-8) were purchased from Dalian Meilun Biotech Co., Ltd (Dalian, China). Anhydrous dimethyl sulfoxide (DMSO, ≥ 99.9%) and S-nitrosoglutathione (GSNO) were purchased from Sigma-Aldrich. 4,5-Diamino-N,N,N',N'-tetraethylrhodamine (DAR-1, ab145388) was purchased from Abcam (Cambridge, MA, USA). Ce6 was purchased from American J&K Scientific Ltd. Griess kit, 4-amino-5-methylamino-2’,7’-difluorofluorescein diacetate (DAF-FM DA), bicinchoninic acid (BCA) protein assay kit, fluorophore-conjugated anti-rabbit secondary antibodies, and 4,6-diamidino-2-phenylindole (DAPI) were purchased from Beyotime Biotechnology Co., Ltd (Shanghai, China). Mouse adenosine enzyme-linked immunosorbent assay (ELISA) kit was obtained from Shanghai Enzyme-linked Biotechnology (Shanghai, China). All other chemicals were obtained from Sinopharm Chemical Reagent Co., Ltd (China).

*Synthesis of SP1:* SP1 was synthesized following a previously reported method.^[^[^1^](#_ENREF_1)^]^ Briefly, the monomer 4,8-dibromo-6-(2-ethylhexyl)-[1,2,5]thiadiazolo[3,4-f]benzotriazole (112 mg, 0.25 mmol) was dissolved in 10 mL of toluene containing 3,6-bis(5-trimethylstannylthien-2-thienyl)-2,5-bis(2-decyltetradecyl)-2,5-dihydropyrrolo[3,4-c]pyrrole-1,4-dione (325 mg, 0.25 mmol) in a 25-mL three-necked flask. The mixture underwent degassing and purging with nitrogen (N_2_) three times after the addition of the Pd(PPh_3_)_4_ (5 mg, 0.004 mmol). The solution was then heated to 100 °C and stirred for 1 day under a N_2_ atmosphere. Gradual dropwise addition of (4,4,5,5-tetramethyl-1,3,2-dioxaborolan-2-yl)benzene (20 mg, dissolved in toluene) and 0.2 mL of bromobenzene was performed separately to remove the end groups. The final solution underwent washing with ultrapure water, purificatopm through Soxhlet extraction, and recrystallization using methanol before evaporation under vacuum.

*Synthesis of SP2:* The synthesis steps of IC-FBr and the monomer (Y-OD-FBr) are similar to previous report.^[^[^2^](#_ENREF_2)^]^ Briefly, Compound **1** (2.63 g, 10 mmol) was dissolved in acetic anhydride (15 mL) and stirred at 140 ^o^C for 3 h. After cooling, triethylamine (8 mL) and tert-butyl acetoacetate (2.0 mL, 13 mmol) were added and the mixture was stirred overnight at 65 °C. The resulting mixture was then processed to isolate compound **3**. **^1^H NMR** (400 MHz, CDCl_3_): *δ* 8.02 (dd, *J* = 8.0, 5.6 Hz, 1H), 7.65 (d, *J* = 8.0 Hz, 1H), 3.28 (s, 2H). **^13^C NMR** (101 MHz, CDCl_3_): *δ* 195.11, 192.36, 155.86, 153.19, 143.78, 141.11, 130.29, 119.89, 119.83, 118.63, 118.43, 45.26. MS (CI) [M] calcd. for (C_9_H_4_BrFO_2_): 241.9379. Found: 241.9373. Compound **3** (0.97 g, 4 mmol) was further reacted with malononitrile (0.53 g, 8 mmol) in 30 mL absolute ethanol with anhydrous sodium acetate (0.66 g, 8 mmol), followed by purification to yield pure **IC-FBr** (0.71 g, 60%). MS (CI) [M] calcd. for (C_12_H_4_BrFN_2_O): 289.9491. Found: 289.9481. Y-OD-CHO (78 mg, 0.05 mmol) and IC-FBr (58 mg, 0.20 mmol) were refluxed in chloroform (15 mL) with pyridine (1 mL), followed by purification to obtain Y-OD-FBr (77 mg, 80%). HR-MS (MALDI-TOF) [M+H]^+^ calcd. for (C_106_H_134_Br_2_F_2_N_8_O_2_S_5_): 1911.7600. Found: 1911.7665. Finally, **Y-OD-FBr** (25.0 mg, 0.013 mmol) and distannylated monomer thiophene-tin (10.6 mg, 0.0258 mmol) were reacted with Pd_2_(dba)_3_ (1.18 mg, 1.29×10^-3^ mmol) and P(*o*-tolyl)_3_ (1.57 mg, 5.16×10^-3^ mmol) in toluene (2 mL) at 110 ^o^C for 3 days. The resulting mixture was processed and purified to yield the final **SP2** (yield: 65%). GPC: Mn = 13.2 kDa, Mw = 29.3 kDa, PDI = 2.21.

*Characterization of Nanoparticles:* UV-vis-NIR absorbance was measured using a Persee spectrophotometer (TU-1810, Beijing, China). Fluorescence spectra were obtained with RF-6000 fluorescence spectrophotometer (Japan) and FLS980 fluorescence spectrometer (Edinburgh, UK). Nuclear magnetic resonance (^1^H NMR) spectra were acquired using a 400 MHz NMR spectrometer (Bruker, Ettlingen, Germany). Particle sizes and zeta potentials were determined using a Zetasizer Nano ZS particle analyzer (Malvern Instruments, Malvern, UK). The nanoparticle morphology was visualized with a transmission electron microscope (TEM, Tecnai, FEI, USA). High performance liquid chromatography (HPLC) analysis was performed on a LC-16 system (SHIMADZU, Japan).

*Cell Culture:* C6 glioblastoma cells stably expressing luciferase (C6-Luc), brain capillary endothelial cells (bEnd.3) and RAW 264.7 macrophages were obtained from Cell Bank, Chinese Academy of Sciences (Shanghai, China). Cells were cultured in DMEM containig 10% FBS and 1% penicillin/streptomycin in a humidified atmosphere.

*In Vivo Evaluation of Biosafety:* To assess the biosafety of nanoparticles, regular monitoring of murine body weights was conducted every 2 days over a 24-day period. H&E staining was perfomed on organs including the heart, liver, spleen, lung, and kidney.

**Reference**

[1] X. Men, F. Wang, H. B. Chen, Y. B. Liu, X. X. Men, Y. Yuan, Z. Zhang, D. Y. Gao, C. F. Wu, Z. Yuan, *Adv. Funct. Mater.* **2020**, 30, 11.

[2] H. Yu, Y. Wang, X. Zou, H. Han, H. K. Kim, Z. Yao, Z. Wang, Y. Li, H. M. Ng, W. Zhou, *Adv. Funct. Mater.* **2023**, 33, 2300712.
